# Betting on the fastest horse: Using computer simulation to design a combination HIV intervention for future projects in Maharashtra, India

**DOI:** 10.1371/journal.pone.0184179

**Published:** 2017-09-05

**Authors:** Kelly V. Ruggles, Anik R. Patel, Stephen Schensul, Jean Schensul, Kimberly Nucifora, Qinlian Zhou, Kendall Bryant, R. Scott Braithwaite

**Affiliations:** 1 Department of Medicine, New York University School of Medicine, New York, NY, United States of America; 2 Department of Experimental Medicine, University of British Columbia Faculty of Medicine, Vancouver, BC, Canada; 3 Department of Community Medicine and Health Care, University of Connecticut Health Center, Farmington, CT, United States of America; 4 Institute for Community Research, Hartford, CT, United States of America; 5 Department of Population Health, New York University School of Medicine, New York, NY, United States of America; 6 National Institute on Alcohol Abuse and Alcoholism, Bethesda, MD, United States of America; Universitatsmedizin Greifswald, GERMANY

## Abstract

**Objective:**

To inform the design of a combination intervention strategy targeting HIV-infected unhealthy alcohol users in Maharashtra, India, that could be tested in future randomized control trials.

**Methods:**

Using probabilistic compartmental simulation modeling we compared intervention strategies targeting HIV-infected unhealthy alcohol users on antiretroviral therapy (ART) in Maharashtra, India. We tested interventions targeting four behaviors (unhealthy alcohol consumption, risky sexual behavior, depression and antiretroviral adherence), in three formats (individual, group based, community) and two durations (shorter versus longer). A total of 5,386 possible intervention combinations were tested across the population for a 20-year time horizon and intervention bundles were narrowed down based on incremental cost-effectiveness analysis using a two-step probabilistic uncertainty analysis approach.

**Results:**

Taking into account uncertainty in transmission variables and intervention cost and effectiveness values, we were able to reduce the number of possible intervention combinations to be used in a randomized control trial from over 5,000 to less than 5. The most robust intervention bundle identified was a combination of three interventions: long individual alcohol counseling; weekly Short Message Service (SMS) adherence counseling; and brief sex risk group counseling.

**Conclusions:**

In addition to guiding policy design, simulation modeling of HIV transmission can be used as a preparatory step to trial design, offering a method for intervention pre-selection at a reduced cost.

## Introduction

HIV simulation models have been successfully used to estimate long-term economic, health and HIV transmission outcomes.[1–4] Historically, these models have been employed to guide HIV policy design by identifying how to apply evidence-based interventions to yield the greatest additional health benefit given available resources. Accordingly, they have informed utilization and targeting of pre-exposure prophylaxis[4], antiretroviral (ART) therapy[5], male circumcision[6] and HIV vaccines.[1,2,7] As there is an increasing focus on combination interventions[8,9], simulations may have an additional role: informing the design of combination interventions trials. More specifically, modeling cases in which the constituent interventions may have been tested independently but have not yet been tested together. A simulation modeling-based approach has the ability to maximize the return on research investment during clinical trial design through the identification of interventions likely to deliver the greatest health benefit for the lowest cost prior to trial initiation. In other words, simulation can be used to identify the horse that is most likely to be fastest (e.g. most likely of having a substantial treatment effect) before betting on it (e.g. expending resources to perform a randomized controlled trial (RCT)).

As in any application of evidence based medicine, computer simulation uses data sources with uncertainty arising from bias, random error, and impaired generalizability when results originate from a different patient population, care system, or region.[10] Nonetheless, this uncertainty can be reflected mathematically by appropriately wide probability distributions that are then propagated through the simulation and are reflected in the level of uncertainty of simulation results.[11] If one strategy appears to be most desirable even after taking uncertainty into account, that strategy is a preferred candidate for additional testing and/or implementation. If no strategies emerge as clearly desirable because the uncertainty is too large, then no clear candidate will be chosen for additional testing and/or implementation, and decisions can be made as per current practice.

With nearly 90% of HIV RCTs failing to demonstrate statistically significant effectiveness[7,12], use of simulation models as a step preparatory to trial design offers a possible method for intervention pre-selection at a limited cost.[13,14] Despite this potential, model-based approaches for HIV trial design have been underused.

The goal of this study was to inform the design of a combination HIV intervention that could be tested in future RCTs. The target patient population are HIV-infected unhealthy alcohol users in Maharashtra, India; possible constituents of the combination intervention to be tested in the modeling study include different target behaviors (unhealthy alcohol consumption, risky sexual behavior, depression, and ART adherence), different formats (individual, group based, community) and different durations (shorter versus longer). To do this, we developed a computer simulation of HIV progression and transmission in Maharashtra, India that could predict clinical outcomes, along with their uncertainty, under 5,386 alternative formulations of a combination intervention, and we investigated whether particular formulations were likely to yield the greatest health benefit given budget constraints and uncertainty of data inputs.

## Methods

### Overview

The established evidence-based framework for formulating clinical questions recommends the question be defined in terms of problem/population, intervention, comparison and outcome, to help clinicians articulate their needs through the generation of focused questioning.[15] In our study, the problem/population was preventing HIV infection in unhealthy alcohol users in Maharashtra, India. Unhealthy alcohol use is the spectrum from risky-users to alcohol use disorders and has been defined by the National Institute on Alcohol Abuse and Alcoholism (NIAAA) as answering yes to the following question “How many times in the past year have you had more than four drinks (for women) or five drinks (for men) in a day?”[16]. Fifteen categories of behavioral interventions were included and our focus was to estimate cost and effectiveness by comparing a combination of the fifteen interventions based on quality-adjusted life years (QALY) gained and intervention costs.

The data and analysis for this paper was collected as a part of the modeling component of the NIAAA-funded, Indo-US research and intervention project entitled, “Alcohol and ART Adherence: Assessment, Intervention and Modeling in India (U01AA021990-01; 2014–2019) based in Mumbai, Maharashtra. The project utilizes a combination of interventions at the individual, group and community levels to reduce drinking and unprotected sex and to increase medication adherence. The role of the modeling component was to estimate the impact of the interventions implemented by this project for HIV-infected men who have consumed alcohol in the last 30 days and to inform current and future stakeholders of interventions for Maharashtra most likely efficient and impactful; this paper focuses on the latter role.

### Simulation modeling

To test these interventions, we developed a simulation that integrates a mechanistic model of HIV prevention and a compartmental model of HIV transmission, both calibrated against India specific epidemiological outcomes (**S1 File**). Both models were based on models previously developed by our group to study HIV progression and transmission in East Africa[2,17,18] and New York City[1].

Efficient frontiers were computed for each of the intervention bundles over twenty-year time horizons to determine strategies delivering the greatest health benefit for the lowest cost.[19] In brief, the efficient frontier was created by calculating the incremental cost-effectiveness ratio (ICER) for a combination of strategies in order to measure the additive benefit of subsequent strategies compared with its next best alternative. In order to account for the error associated with model inputs we used probabilistic modeling to calculate model inputs and intervention cost and effectiveness measures based on variable-specific probability distributions[20].

### HIV natural history model

HIV progression was modeled using a stochastic simulation model, in which disease progression was simulated for 100,000 patients, tracking CD4 and HIV-1 viral load (VL) transitions based on antiretroviral treatment and drug adherence. This model is based on previous validated models of HIV progression[17,21], with updated India-specific parameters (**S1 Table**) and has been recalibrated to epidemiological data from Maharashtra, India (**S1 Fig**). The natural history model takes into account non-adherence as the primary cause of ART failure and drug resistance, although resistance can arise with lower probability even when ART adherence is favorable.[18]

Output distributions from this model were collapsed into rate multipliers, which were subsequently used in our HIV transmission simulation model to determine rates of movement through CD4 strata (<50, 51–200, 201–350, 351–500, >500 cells/mm^3^), logarithmic VL strata (<2.5, 2.5–3.5, 3.5–4.5, 4.5–5.5, >5.5 log copies/ml) and HIV status (initial infection, undetected, detected, in care, on treatment, dead) (**S1 Fig**).

### HIV transmission simulation model

An SIR-compartmental model of HIV transmission was developed in C++ programming language as described previously[2] and modified to better represent the population of India including compartments for injection drug use (IDU) and sexual orientation, in addition to the use of India specific model input parameters. Hypothetical people in the model occupy one set of mutually exclusive and collectively exhaustive compartments at all times and as time proceeds these hypothetical people may change the compartment they occupy. In addition to clinical characteristics specific to HIV (HIV status, VL, CD4), the compartments of this model include age, four sexual activity levels (abstinent, monogamous, multiple partnerships, commercial sex workers (CSWs) and their clients), unhealthy alcohol status, IDU status and sexual orientation (**S2 Fig**).

Both sexual transmission and transmission through IDU needle sharing are modeled, and assumed to follow heterogeneous mixing in the population.[2,22] The probability of HIV transmission between partners is based on gender of both the infected and non-infected partner, and the disease and treatment status of the infected partner (**S2 Fig**).

### HIV clinical trial interventions

Our HIV transmission simulation model is able to represent the implementation of one or a combination of intervention strategies. These interventions act on pathways that reduce HIV transmission[1] (**S1 File**), specifically pathways affecting alcohol use, condom use prevalence, STI prevalence, depression and ART adherence. Unhealthy alcohol use was modeled as increasing the relative risk of condom nonuse (RR = 1.29), STI prevalence (RR = 1.72) and the risk of ART non-adherence (RR = 2.33) and therefore interventions targeting the alcohol use pathway impact all three of these risk behaviors.[2] Depression and adherence interventions were modeled as reducing the risk of ART non-adherence[23,24] and interventions acting on sex risk acted through reducing condom nonuse and STI prevalence.[25,26] At the time of model development we found no existing evidence in the literature for a direct impact of adherence or depression on condom use or STI prevalence and therefore did not include an associated effect through these pathways. Effect Relative Risk (RR) and 95% confidence intervals specific for each intervention and behavior pathway can be found in **Table 1**.

**Table 1:** Clinical trial interventions and associated costs and effects considered in HIV transmission simulation model. Uniform distributions were used for all costs and lognormal distribution for all effects in probabilistic analyses. Intervention costs were derived from India-specific sources.[35,49–51] Cost in 2012 USD.

Intervention	Risk pathway	Effect RR (95% CI)	Session Length (Hours)	Cost (Range)	RR Ref.
Alcohol: Brief individual counseling	Alcohol use	0.68 (0.50–0.93)	1	$1.64 (0.5x-1.5x)	[27]
Alcohol: Long individual counseling	Alcohol use	0.36 (0.15–0.82)	4	$6.56 (0.5x-1.5x)	[27]
Alcohol: Brief group counseling	Alcohol use	0.62 (0.42–0.91)	5	$1.64 (0.5x-1.5x)	[27]
Alcohol: Long group counseling	Alcohol use	0.47 (0.25–0.86)	18	$5.90 (0.5x-1.5x)	[27]
Sex risk: Brief individual counseling	Condom use	0.97 (0.95–0.99)	1	$1.64 (0.5x-1.5x)	[25]
	STI prevalence	0.84 (0.73–0.96)			
Sex risk: Long individual counseling	Condom use	0.92 (0.85–0.98)	9	$14.76 (0.5x-1.5x)	[25]
	STI prevalence	0.64 (0.44–0.89)			
Sex risk: Brief group counseling	Condom use	0.97 (0.94–0.99)	5	$1.64 (0.5x-1.5x)	[25]
	STI prevalence	0.81 (0.68–0.95)			
Sex risk: Long group counseling	Condom use	0.94 (0.90–0.99)	18	$5.90 (0.5x-1.5x)	[25]
	STI prevalence	0.71 (0.54–0.92)			
Sex risk: Community intervention	Condom use	0.97 (0.94–0.99)	ongoing	$6.66 (0.5x-1.5x)	[26]
	STI prevalence	0.78 (0.59–1.0)			
Depression: Brief individual counseling	Depression	0.84 (0.43–1.0)	8	$13.12 (0.5x-1.5x)	[28]
Depression: Long individual counseling	Depression	0.62 (0.28–1.0)	22	$36.08 (0.5x-1.5x)	[28]
Depression: Brief group counseling	Depression	0.81 (0.65–0.97)	11	$3.61 (0.5x-1.5x)	[28]
Depression: Long group counseling	Depression	0.71 (0.58–0.84)	22	$7.22 (0.5x-1.5x)	[28]
Adherence: Brief counseling	ART Adherence	0.67 (0.53–0.84)	1	$6.56 (0.5x-1.5x)	[24]
Adherence: Weekly SMS	ART Adherence	0.75 (0.58–0.96)	4	$2.46 (0.5x-1.5x)	[24]

A compartmental model approach was required to address this particular problem, as opposed to back-of-the-envelope calculations, because of the large number of plausible choices, other than the obvious expectation that a multi-level intervention aimed at alcohol users would contain an alcohol intervention of some kind. Nonlinear interactions in the model included effects of alcohol and depression on adherence, in accord with the cited literature.

Fifteen behavioral interventions were included, four specifically addressing unhealthy alcohol use, five addressing sex risk behavior, four addressing depression and two addressing ART adherence (**Table 1**). Target behaviors were chosen based on prior qualitative research, which suggested that these were the most prevalent behavioral risk factors for HIV in this population. Interventions targeting unhealthy alcohol use, depression and sex risk were split into four subgroups: Brief individual counseling; Long individual counseling; Brief group counseling; and Long group counseling. The length of these sessions was determined using published meta-analysis specific to each intervention type and can be found in **Table 2**.[24–28] Alcohol interventions in this study refer to motivational interviewing for the prevention of alcohol misuse.[27] Following the completion of our analysis, this study (Foxcroft et al.)[27] was withdrawn due to errors in the analysis. However, an updated meta-analysis by the same group was published in 2016, summarizing the effect of motivational interviewing on reducing alcohol use in young adults, now including an additional 18 randomized trials[29]. The results of this meta-analysis were consistent and not statistically different from those reported in the original study, and were within the confidence interval input included in our probabilistic analysis.

**Table 2:** India transmission model inputs.

Model Parameter	Value	Probabilistic range	Distribution	Ref.
Age of sexual debut	19	17–22	uniform	[31]
**Sexual risk characteristics**				
Proportion of men who are homosexual	0.0006	0.0006–0.0097*	uniform	[30]
Proportion of men who are bisexual	0.0004	0.0004–0.00049	normal	[30]
Proportion women who are homosexual	0.0006	—	—	*
Proportion of women who are bisexual	0.0004	—	—	*
**Proportion abstinent**				
Straight males	0.2775	0.2241–0.4260	normal	[34]
Homosexual/Bisexual males	0	—	—	-
Straight/Homosexual/Bisexual females	0.260	0.1227–0.4378	normal	[31]
**Proportion monogomous**				
Straight males	0.573	0.3857–0.6616	normal	[34]
Homosexual/Bisexual males	0.230	0.1541–0.3035	normal	[30]
Straight/Homosexual/Bisexual females	0.690	0.5345–0.8478	normal	[31]
**Proportion concurrent partners**				
Straight males	0.1292	0.068–0.194	normal	[34]
Homosexual/Bisexual males	0.675	—	—	-
Straight/Homosexual/Bisexual females	0.047	0.0199–0.0273	normal	[34]
**Proportion CSW or clients of CSW**				
Straight males	0.02	0.02–0.2*	uniform	[32]
Homosexual/Bisexual males	0.095	0.0744–0.1198	normal	[30]
Straight/Homosexual/Bisexual females	0.003	0.003–0.03*	uniform	[30]
**Average duration (years) of partnership**				
Monogomous	30.0	0.5x-1.5x	uniform	[2]
Concurrent partners	1.0	0.5x-1.5x	uniform	[2]
CSW or clients of CSW	0.5	0.5x-1.5x	uniform	[2]
**Median number of concurrent partnerships**				
Monogomous	1	—	—	[59]
Concurrent partners	3	0.5x-1.5x	uniform	[59]
CSW or clients of CSW	10	0.5x-1.5x	uniform	[59]
Degree of assortative mixing	0.2	0.05–0.5	uniform	[60]
**HIV risk behavior modifiers**				
Prevalence of unhealthy alcohol use: females	0.020	0.0096–0.0244	normal	[61]
Prevalence of unhealthy alcohol use: males	0.185	0.0925–0.2775	uniform	+
Proportion of unhealthy alcohol users who are depressed	0.57	—	—	**
Proportion of condom nonuse	0.73	0.730–0.815	normal	[31]
Probability of not being tested for HIV	0.98	0.960–0.990	uniform	[33]
Probability of ART nonadherence	0.26	0.260–0.364	normal	[62]
Probability of untreated STI	0.06	0.060–0.096	normal	[63]
**Sexual and IDU transmission**				
Transmission risk per sex act				
Male to Male	0.00167	0.5x-1.5x	normal	[64]
Female to Male	0.00081	0.5x-1.5x	normal	[64]
Male to Female	0.00042	0.5x-1.5x	normal	[64]
Transmission risk per unsafe needle sharing	0.0036	0.5x-1.5x	normal	[65]
Relative risk of transmission if viral load:				[66]
0–2.5	0.16	—	—	
2.5–3.5	1.87	—	—	
3.5–4.5	6.54	—	—	
4.5–5.5	8.85	—	—	
>5.5	9.03	—	—	
**Injection Drug Use Characteristics**				
Proportion of population that injects drugs	4.99E-05	4.99E-5-0.0025*	uniform	[30]
Number of needle sharing partners per year	5	0.5x-1.5x	uniform	[67]
Shared injections per year	102	54–150	uniform	[30]
**HIV disease related**				
CD4 Mean	644	294–994	uniform	[35]
CD4 SD	260	65–585	normal	[35]
VL Mean	4.46	4–5	uniform	[35]
**Utility**				
CD4<50	0.79	0.74–0.84	uniform	[68]
CD4 51–200	0.85	0.8–0.9	uniform	[68]
CD4 > 201	0.94	0.89–0.99	uniform	[68]
Change with ART treatment	-0.053	0.5x-1.5x	uniform	[68]

*Due to lack of evidence on these variables, we made assumptions on point estimates and included broad distribution ranges to compensate for our lack of knowledge in uncertainty analysis.

+Based on survey responses from our patient population (unpublished)

**Based on personal communication with Dr. Jean Schensul

Interventions to reduce depression correspond to cognitive-behavioral interventions focused on mood and anxiety disorders in an HIV-infected population.[28] Sex risk interventions at an individual and group level in our model were based on the effectiveness of behavioral interventions to increase condom use and reduce sexually transmitted infections (STIs).[25] ART adherence targeting strategies were split into two subgroups: Brief counseling and Weekly Short Message Service (SMS) based on available intervention literature. A community level intervention targeting sexual risk was also included, modeling population-wide condom promotion and/or distribution that was ongoing through the modeling time horizon (**Table 1**).[26]

Within the model, interventions were applied to only males with unhealthy alcohol use with the exception of the community level sex risk intervention, which was applied to the entire population. Efficacy data for interventions were derived from published meta-analysis (see **S1 File** for additional details).[24–28] Intervention costs were assumed to consist of labor and program costs, accounting for administration, overhead and management. Labor costs were calculated as a function of estimated intervention time, also derived from meta-analysis studies.

### Model inputs

Our initial population was calculated using census data from Maharashtra, India from 1991 and 2001, interpolating to derive an estimate for 1997 reflecting both the HIV-uninfected and HIV-infected population. The model population was then divided into population compartments based on gender, sexual risk behavior, sexual orientation (straight, homosexual or bisexual), infection status, treatment status, IDU and unhealthy alcohol use. India-specific sources were then used to determine the initial proportion allocated to each compartment and inputs to the model, the majority of which came from state-level data from Maharashtra collected by HIV clinics between 2007–2014.[30–33] Sexual risk was categorized as abstinent, monogamous, multiple concurrent partners, or as community sex workers (CSW) and their clients.[30,32,34] Although included in our model for completeness, the proportion of the population who identified as homosexual, bisexual or IDU was low (<0.0009), and was therefore not a major contributing factor in HIV transmission. For the HIV-infected subset of our modeled population the mean CD4 was 644 cells/mm^3^ and mean VL was 4.46 log.[35] All inputs were attained from peer reviewed or surveillance literature specific to India or through agreement amongst the study team (see **Table 2**and **S1 Table** for exhaustive model inputs). Several variables were unavailable specifically for India (average duration of partnerships, transmission risk parameters, HIV disease related characteristics) and values that were previously used in our East Africa[2,17,18,36] or New York City[1,37] models were carried through with the assumption that these were not country specific.

#### Alcohol-related parameters

Based on a systematic review of pathways through which alcohol may impact HIV transmission risk in unhealthy alcohol use was modeled as having three main effects: (1) increasing the risk of condom nonuse (RR 1.29 for unsafe sex [38,39]) (2) increasing the risk of ART non-adherence (RR 2.33 of missing doses based on pooled estimate from 4 studies [40–43]) and (3) increasing sexually transmitted infection (STI) prevalence (RR 1.72). [44,45] There were several limitations in these parameters due to data availability. For all the risk parameters, India-specific variables were unavailable, despite an extensive review of the literature. For this reason, we used data from several comprehensive Africa studies which has been successfully used to model the impact of alcohol interventions on HIV transmission in East Africa[36], with the assumption that the impact of alcohol on behavior is universal and the country-specific variability would be taken into account by our probabilistic analysis.

Exploring the impact of each intervention bundle on key model compartments, including clinical outcomes of untreated HIV, HIV status and alcohol use has been visualized in **S4 Fig**.

### Validation

Four validation criteria (HIV prevalence, incidence, the proportion of people with HIV on treatment and the annual proportion of people who have died from HIV) were used to test the accuracy of the model predictions compared with epidemiological data from Maharashtra, India between 1997 and 2013.[46] Comparison of our simulation results with these data demonstrated reasonable goodness of fit with HIV prevalence, incidence, proportion of people with HIV on treatment and the annual proportion of people who have died from HIV (**S3 Fig**).

### Cost-effectiveness analysis

Clinical outcomes of this model include the number of infections averted, number of AIDS-related deaths averted and the number of Quality Life Years (QALYs) across the population for a 20-year time horizon. We used efficiency frontiers to identify intervention packages delivering the largest health benefit within a specified budget constraint across the 20 year time horizon.[47] Strategies outside the frontier are unfavorable options, as they are unable to deliverer the largest benefit at any budget level. Efficient frontiers were determined by calculating the incremental cost-effectiveness ratio (ICER) of intervention packages, which measure the additive benefit of each intervention strategy compared with its next best alternative. This calculation starts with a control simulation, in which there are no interventions applied, and one intervention simulation. The ICER is then calculated as difference in total costs between the intervention package(s) and the control group divided by the difference in QALYs observed between intervention and control. Additional interventions are then added, and an efficient frontier is calculated using standard methods (see Gold et al.[48] for a comprehensive review). The efficient frontier cumulatively takes into account the difference in incremental cost-effectiveness ratios between alternative interventions, and allows for the determination of the most effective intervention at different budget price points.

Costs and QALYs were discounted at 3%, and costs were assessed in 2014 US dollars. We estimated weighted utility scores (used in the calculation of QALYs) based on data from HIV-infected persons in the developed world. Intervention cost inputs were based on data from several India-specific sources.[35,49–51]

### Probabilistic uncertainty analysis

**Fig 1:**
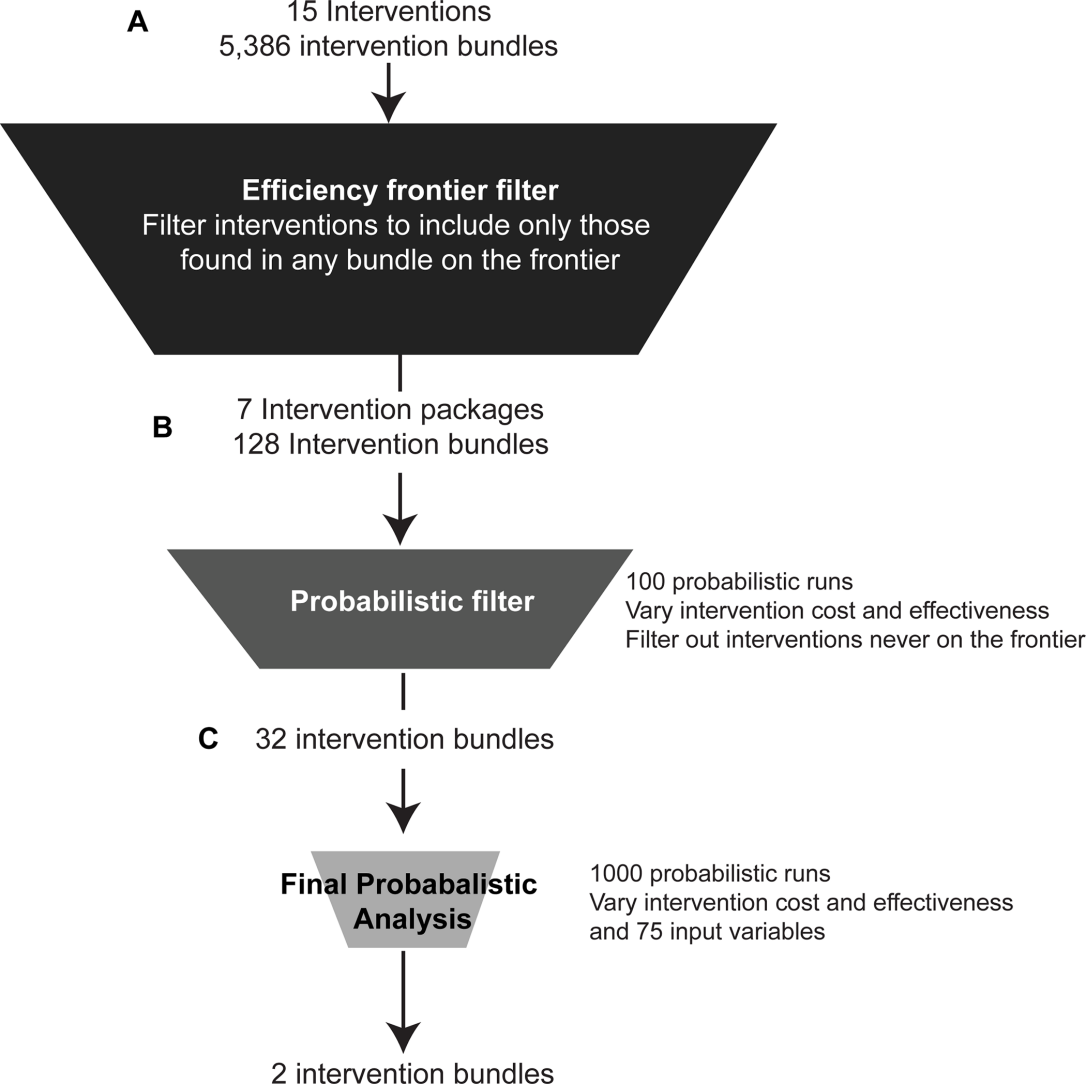
Analyses methodology. Pipeline workflow for intervention bundle prioritization. a, Creation of efficient frontier for all combinations of 15 interventions and filtering out 8 interventions that were never found on the frontier. b, For the remaining 7 interventions, completion of 100 probabilistic runs varying intervention costs and effects and filtering out intervention bundles that were never found on the frontier. c, Completion of a full probabilistic analyses (run N = 1000) varying intervention cost and effect as well as 96 input variables. All analysis was run for a 20-year simulation.

We first examined unique and logically consistent permutations of the 15 constituent interventions, which in combination resulted in 5,386 possible intervention bundles ranging from individual interventions to a combination of 9 interventions simultaneously (**Fig 1A**). The analysis was then completed by running twenty-year simulations of each intervention bundle using base-case input values. Bundles appearing on the efficient frontier were carried through to the next screening step.

Our uncertainty analysis was done in two stages and used a 95% confidence intervals (where possible) and a wide range of values when evidence was limited. In the initial stage we incorporated parameter uncertainty to the cost and effectiveness of each intervention as a screening procedure for the second stage (**Fig 1B**). Interventions identified based on our initial deterministic analysis were run in twenty year simulations 100 times using probabilistically varied cost and effect values for each intervention. Uniform distributions were used for all costs and lognormal distributions for all effects. The upper and lower limits of these parameter inputs can be found in **Table 1**.

In the second stage, definitive probabilistic analysis was then completed on a more inclusive variable list to assess the robustness of each intervention given a certain level of uncertainty (**Fig 1C**). Intervention bundles found to be on the frontier at least once in the screening probabilistic stage were run in a full probabilistic analysis (N = 1000), varying intervention cost and effect, and 96 input variables related to population parameters, HIV transmission, and treatment parameters (**Table 2, S4 Fig**). Sampling of each variable was done using specified limits and distribution types simultaneously (**S1 Table)**. We used a robustness cutoff of 50% or more of runs being on an efficient frontier in choosing bundles likely to yield the greatest health benefit for the resources consumed.

For both stages of probabilistic analysis, intervention simulation runs were compared against matched control runs containing the same set of cost and effectiveness parameters (stage 1 and 2) and input variables (stage 2 only) based on the same randomly generated number seed. Each random pull from the distributions was unique, so the simulations can be thought of as 100 (stage 1) or 1000 (stage 2) independent clinical trials, each with a matched control (no intervention) run. The distributions of clinical outcomes (discounted costs and QALYs, new infections and deaths, etc.) resulting from the varied input variables under control conditions can be visualized in **S7 Fig**.

Code and technical appendices for both the progression and transmission model can be found here https://github.com/braithwaitelab/india.

## Results

### Identifying best combination interventions based on deterministic analysis

**Fig 2:**
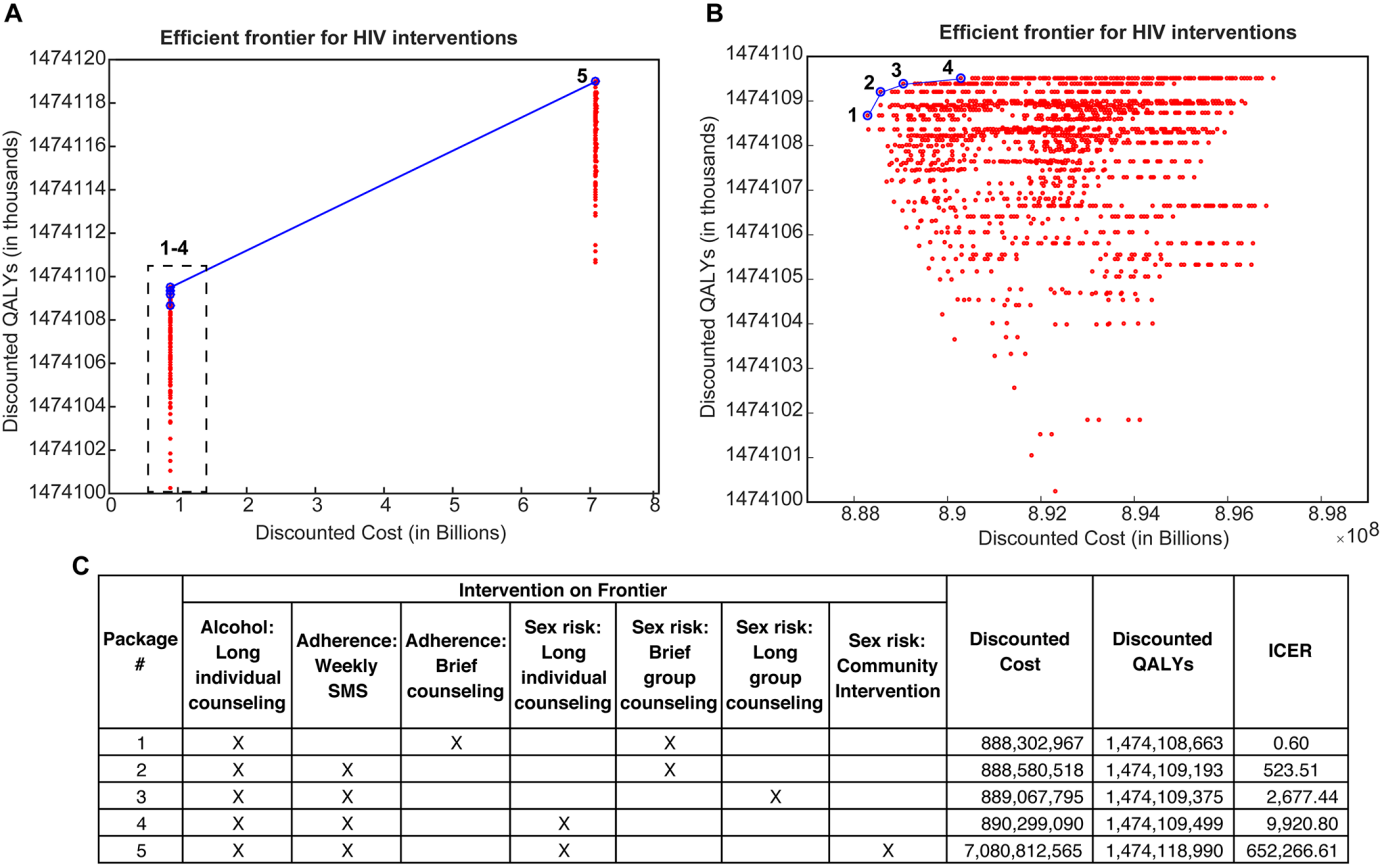
Efficient frontier for HIV interventions during a 20-year simulation of HIV epidemic in Maharashtra, India. a, Graphical representation efficient frontier for all permutations of 12 interventions (4096 total combinations). Blue circles represent packages of interventions on the frontier, red represent packages off the frontier. b, focused graphical representation of efficient frontier for the lower end of discounted cost (0.888–0.898 Billion USD). c, Interventions contained within each efficient frontier package.

Based on twenty-year simulations of 5,386 combinations of the 15 interventions (**Fig 1A**) we identified 7 interventions appearing on the efficient frontier either alone or as part of a combined intervention bundle (**Fig 2A and 2B**), meaning that health benefit was maximized given the resources expended. These 7 intervention candidates were Alcohol: Long individual counseling, Adherence: Weekly SMS, Adherence: Brief counseling; Sex risk: Long individual counseling, Sex risk: Brief group counseling, Sex risk: Long group counseling, and Sex risk: Community intervention (**Fig 2C**). Eight interventions were never found on the efficient frontier in any combination bundle.

It should also be noted that although the change in discounted QALYs between the most and least effective intervention packages is just under 20,000, the target population of male HIV infected unhealthy alcohol users was less than 0.0001% (~1305 out of over 99 million) of the population at the start of the simulation. Therefore, in the context of such a small target population, this seemingly small QALY increase is clinically relevant.

### Probabilistic analyses

The 7 constituent interventions yielded 128 possible formulations of combination interventions that could achieve the greatest health benefit given resources consumed. In order to assess whether any of these bundles were sufficiently robust to anchor an efficient frontier we completed a stochastic analysis where we probabilistically varied intervention cost and effect values for each intervention across 100 runs, as a pre-screening step for the final analyses (**Table 1**, **Fig 1B**).

These runs identified only 32 intervention bundles as ever being on the efficient frontier, twelve of which were found on the frontier more than 10% of the time (**S6 Fig**). The bundle found on the frontier most frequently was a combination of 3 interventions: Alcohol: Long individual counseling; Adherence: Weekly SMS; and Sex risk: Brief group counseling, which was identified on the frontier in 63 of the 100 runs. The 32 bundles identified in this analysis were assigned bundle numbers (1–32) for subsequent analysis steps.

**Fig 3:**
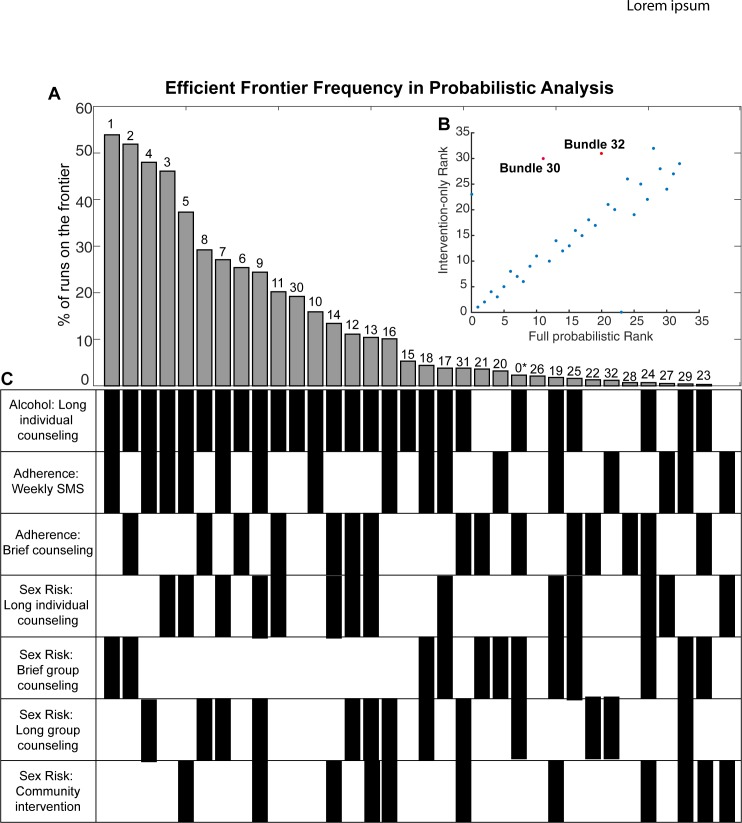
Final probabilistic analysis of top 32 intervention bundles. a, Percentage of runs in which each bundle was identified on the efficient frontier across 1000 probabilistic runs using the 32 bundles identified in the previous analysis step. b, bundle ranking comparison between intervention-only probabilistic and the full probabilistic analysis. c, intervention bundle details corresponding to panel a. *Bundle 0 represents runs with no intervention.

These 32 intervention bundles were then run in a definitive probabilistic analysis (N = 1000), varying intervention cost and effect, and 96 input variables related to population parameters, HIV transmission, and treatment parameters (**S5 Fig**). **Fig 3**shows the results of twenty-year simulations across these 1000 probabilistic runs. Based on our criteria for robustness (more than 50% of runs being on an efficient frontier) two consistent bundles were likely to yield the greatest health benefit for the resources consumed, even after considering data uncertainty. These were Bundle 1 (Alcohol: Long individual counseling; Adherence: Weekly SMS; Sex Risk: Brief group counseling) and Bundle 2 (Alcohol: Long individual counseling; Adherence: Brief counseling; Sex Risk: Brief group counseling). Two others came notably close to our 50% criterion, Bundle 3 (Alcohol: Long individual counseling; Adherence: Weekly SMS; Sex Risk: Long group counseling) and Bundle 4 (Alcohol: Long individual counseling; Adherence: Weekly SMS; Sex Risk: Long individual counseling) (**Fig 3**).

## Discussion

This study showed that simulation modeling in combination with probabilistic uncertainty analysis is a powerful tool in the pre-selection of HIV clinical trial interventions. This methodology allows for more robust predictions than typical sensitivity analysis, which only address variation in input values. To our knowledge, this is the first study using dynamic transmission modeling with the primary goal of informing clinical trial design and other studies of comparative effectiveness. In 2014, Cuadros et al. showed the potential utility for agent-based Monte Carlo models in HIV trial design[13] and our study has built upon this premise, now taking into account uncertainty in transmission, demographics, and intervention cost and effectiveness for trial optimization. Further, while probabilistic sensitivity analysis varying transmission parameters in addition to clinical and economic inputs have been used to evaluate the effectiveness of clinical interventions (particularly in the context of influenza vaccination[52–54])[53]^53525153525150424140^, the majority of cost-effectiveness analyses have used a static modeling approach.

Although this proof-of-concept study was designed in the context of the HIV epidemic in Maharashtra, India, the results of this simulation can likely be expanded into other states in India, particularly since many of the variables which we were unable to find specifically for Maharashtra were collected at the country level. Additionally, our probabilistic method covers a large range of population and intervention characteristics that can account for state-specific and, in cases of other lower middle-income countries (LMIC), region-specific differences. In instances where input variables differ significantly by region, recalibration and reanalysis using updated model inputs would be feasible and straightforward. For example, Uzbekistan and Vietnam represent good candidates for repurposing of our model, as they have similar demographics to India based on median age (27.6–30.1 years), birth rates (15.7–19.3 births/1000 population), HIV prevalence (0.15–0.48%), urbanization (32.7–36.4% of total population) and alcohol use (4.3–6.6 liters per adult per capita year).[55,56] Alternatively, European countries such as Ukraine and Armenia and Sub-Saharan countries such as Kenya and Zambia differ significantly from India would likely require the construction of a region-specific model.

In this specific example, our two-stage probabilistic uncertainty analysis identified two intervention bundles that were found on the efficient frontier in more than 50% of the runs, and two that were just below this robustness criteria cutoff. All four of the most promising intervention bundles consisted of three interventions each. All included the Alcohol: Long individual counseling intervention, one of the two adherence interventions (Brief counseling or Weekly SMS) and varied most in the Sex Risk intervention type (**Fig 3A**). Of the four counseling types included in the analysis, depression counseling was never found to be part of a preferred intervention bundle. This is due to the relatively high cost of depression counseling compared to other counseling types included in the model (e.g. $13.12 for brief depression counseling compared to $1.64 for both brief alcohol and sex risk counseling). We expect that if the associated cost was substantially reduced this intervention would be found as part of the best performing bundles more often. Our recommendation, given these results, would be to design a trial with at least two intervention arms containing the two most robust intervention bundles (Bundle 1: Alcohol: Long individual counseling; Adherence: Weekly SMS; Sex Risk: Brief group counseling; and Bundle 2: Alcohol: Long individual counseling; Adherence: Brief counseling; Sex Risk: Brief group counseling), with the potential for a four intervention arm study containing the four highest ranking intervention packages, dependent on monetary and logistical constraints.

There were several limitations to this study. For one, we were unable to account for all possible uncertainty, due in part to computational constraints. For example, the stochastic screening step filtered out 96 bundles never found on the efficient frontier when varying intervention cost and effectiveness, reducing the number of bundles run in the definitive probabilistic analysis from 128 to 32 (**Fig 1B**). However, we were able to test 1000 probabilistic samplings for nearly 100 variables in these 32 robust intervention bundles. Additionally, in nearly all instances our full probabilistic analysis agreed with the intervention-only probabilistic test, in terms of bundle ranking, with the exception of Bundle 30 (Alcohol: Long individual counseling) and Bundle 31 (Alcohol: Long individual counseling; Adherence: Brief counseling; Sex risk: Long group counseling; and Sex Risk: Community Intervention) (**Fig 3B**), giving confidence to our stochastic screening step. The criteria for robustness cutoff of 50% was arbitrary and two bundles came up just under this constraint. Our simulation does not account for possible effects of unhealthy alcohol use on HIV progression outside of its effect on ART non-adherence. Further, there were limitations in our conceptualization of the interventions, as they were all based on meta-analysis of multiple studies. Additionally, it is unclear at this point what level of uncertainty is enough to build into probabilistic transmission models. Standard statistical models estimate parameters as point estimates with variation in terms of 95% confidence intervals or standard errors. Best Practices developed by Good Research Practices in Modeling Task Force recommends use of confidence intervals in all uncertainty analysis when available and broad estimates when evidence is scarce.[57] However, Best Practices in the context of dynamic transmission model have not yet been established.[53]

It may be argued that this analysis strategy should only be performed in the context of a full Value of Information analysis[58] that would consider trial costs in addition to intervention costs, and would allow for the possible result that no trial should be performed at all (that is, some or no interventions should be offered even without additional information). However, simulation analysis can and should be used to inform the effectiveness likely to be observed in a clinical trial. Our analysis does not automatically imply that such a trial would indeed show effectiveness, nor that a trial is necessary to justify implementation of the intervention. Future work may include extending our analysis to estimate Net Monetary Benefit of alternative trial design choices, including the possibility that no trial should be performed at all. Such an analysis would not only be of theoretical interest, but may inform the spectrum of possible decisions considered by stakeholders and funders in the future.

In conclusion, our approach to probabilistic stochastic modeling in HIV transmission allows for upstream assessment of intervention selection at a reduced cost and can be used to inform future iterations of both trial and policy design of multi-level interventions to reduce HIV transmission.

## Supporting information

S1 Fig Calibration of India-specific HIV progression model. a, Comparing model generated survival curve with administrative data from India. b, Comparing model generated time to treatment failure with reported data for India. c, Comparing model generated CD4 recovery after ART initiation to administrative data from India.
(PDF)

S2 Fig HIV transmission model schematic. (PDF)

S3 Fig Validation of HIV epidemic model. a, Comparing model prevalence results with reported data for India. b, Comparing model incidence results with reported data for India. c, comparing annual proportion of people dying of HIV in model with reported data for India. d, comparing proportion of people with HIV on treatment compared with reported data for India.
(PDF)

S4 Fig Compartment dynamics in probabilistic intervention simulations. Mean % change in key model compartments at 20 years for top intervention bundles compared to baseline (no intervention) control across 1000 probabilistic runs. Color legends were split to allow for easier visualization. Color legend 1 corresponds to the first 3 variables and color legend 2 for the last 39.
(PDF)

S5 Fig Histograms of probabilistic analysis for key input variables. (PDF)

S6 Fig Intervention cost and effectiveness probabilistic analysis. a, Graphical representation of intervention bundles identified on the efficient frontier across 100 probabilistic runs of all combinations of 7 interventions. b, focused for lower end of discounted cost (0.888–0.893 Billion USD). c, intervention bundle details corresponding to a and b.
(PDF)

S7 Fig Distribution of epidemic outcomes across 1000 probabilistic baseline runs after 20 year simulations. a., Discounted cost of care and treatment (2014 USD), b., total discounted QALYs for all and c., HIV infected and HIV infected in treatment, d., the number of new infections and HIV infected deaths over 20 years and e., the mean number of new infections and HIV deaths per infected per year.
(PDF)

S1 Table Key model inputs. (PDF)

S1 File Technical Appendix. (DOCX)
