# Supplementary material for: Betting on the fastest horse: Using computer simulation to design a combination HIV intervention for future projects in Maharashtra, India
Source: PLoS One. 2017 Sep 5;12(9):e0184179. doi: 10.1371/journal.pone.0184179 (PMC5584966; doi:10.1371/journal.pone.0184179)
Supplement: S1 Fig — a, Comparing model generated survival curve with administrative data from India. b, Comparing model generated time to treatment failure with reported data for India. c, Comparing model generated CD4 recovery after ART initiation to administrative data from India. (PDF) [file pone.0184179.s001.pdf]

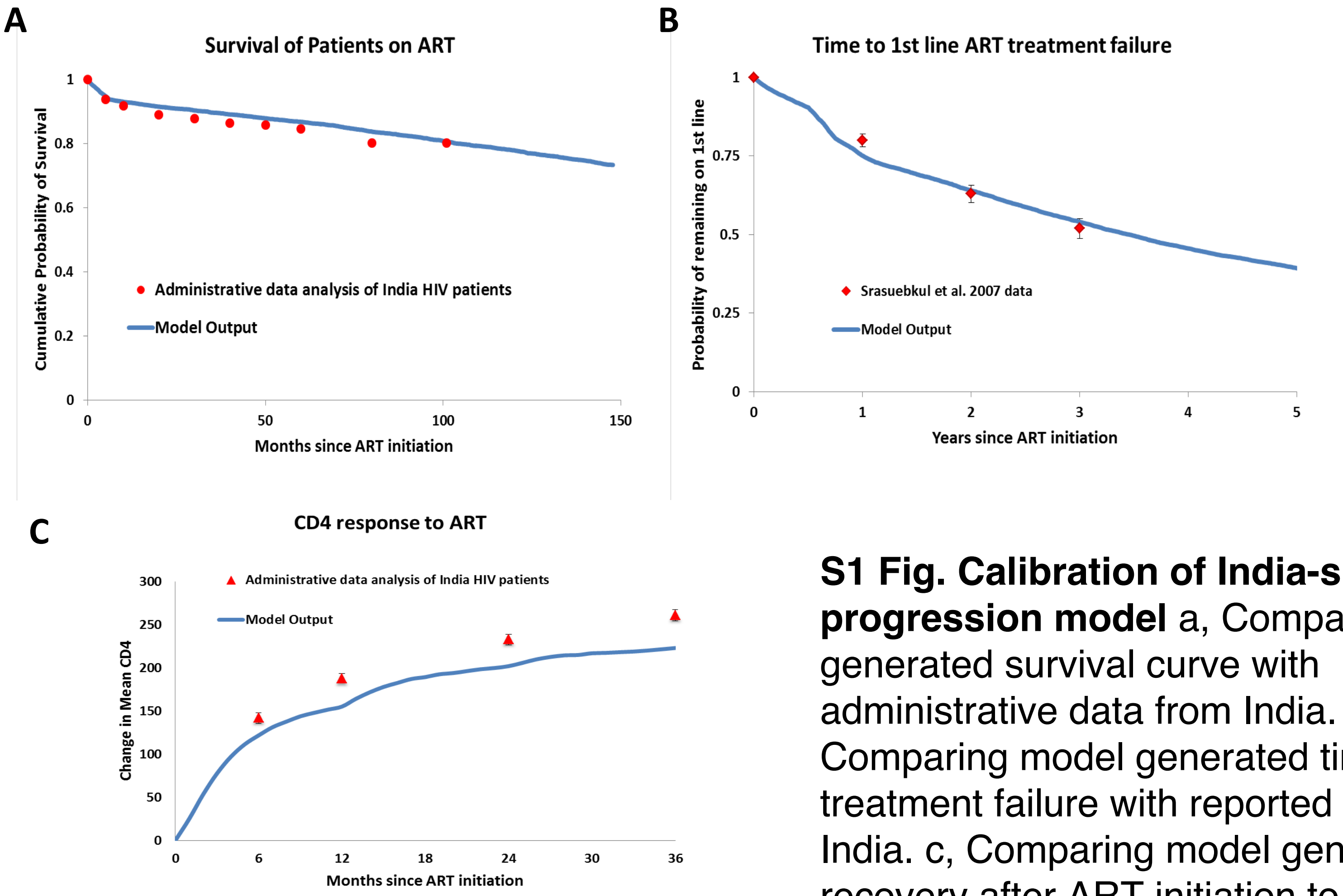

S1 Fig. Calibration of India-specific HIV progression model a, Comparing model generated survival curve with administrative data from India. b, Comparing model generated time to treatment failure with reported data for India. c, Comparing model generated CD4 recovery after ART initiation to administrative data from India.
