# Supplementary figures and images for: Betting on the fastest horse: Using computer simulation to design a combination HIV intervention for future projects in Maharashtra, India
Source: PLoS One. 2017 Sep 5;12(9):e0184179. doi: 10.1371/journal.pone.0184179 (PMC5584966; doi:10.1371/journal.pone.0184179)

HIV Natural History Simulation

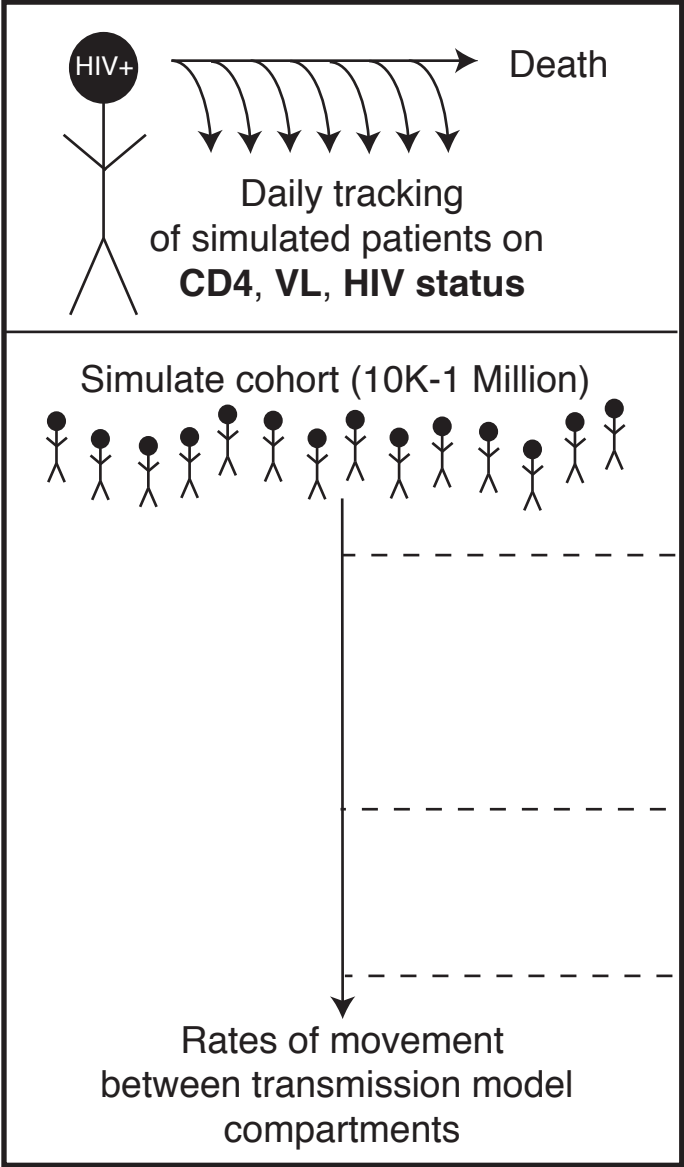

HIV Transmission Model

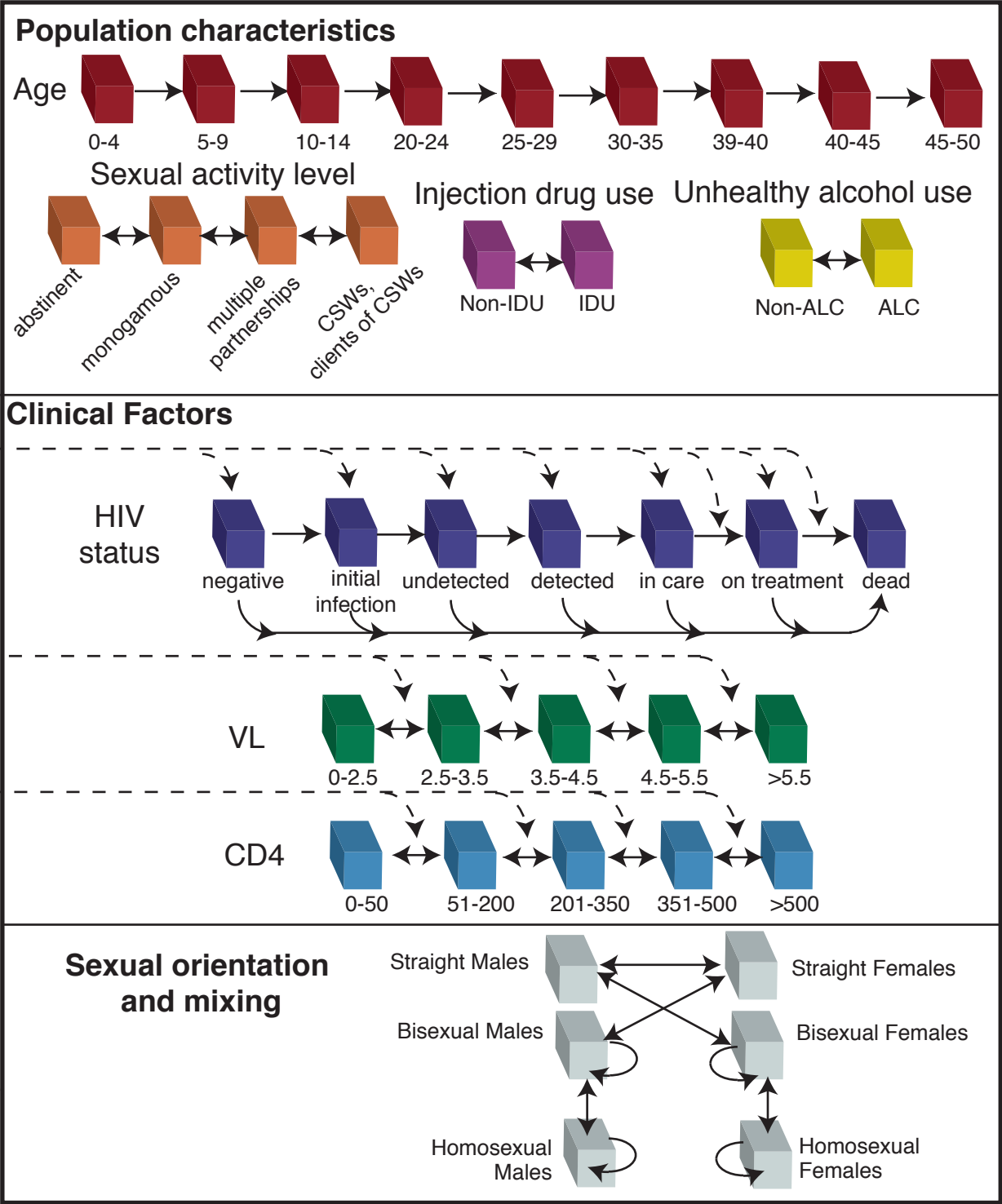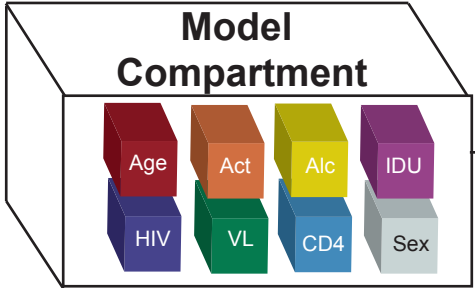

S2 Figure

Supplement: S2 Fig — (PDF) [file pone.0184179.s002.pdf]

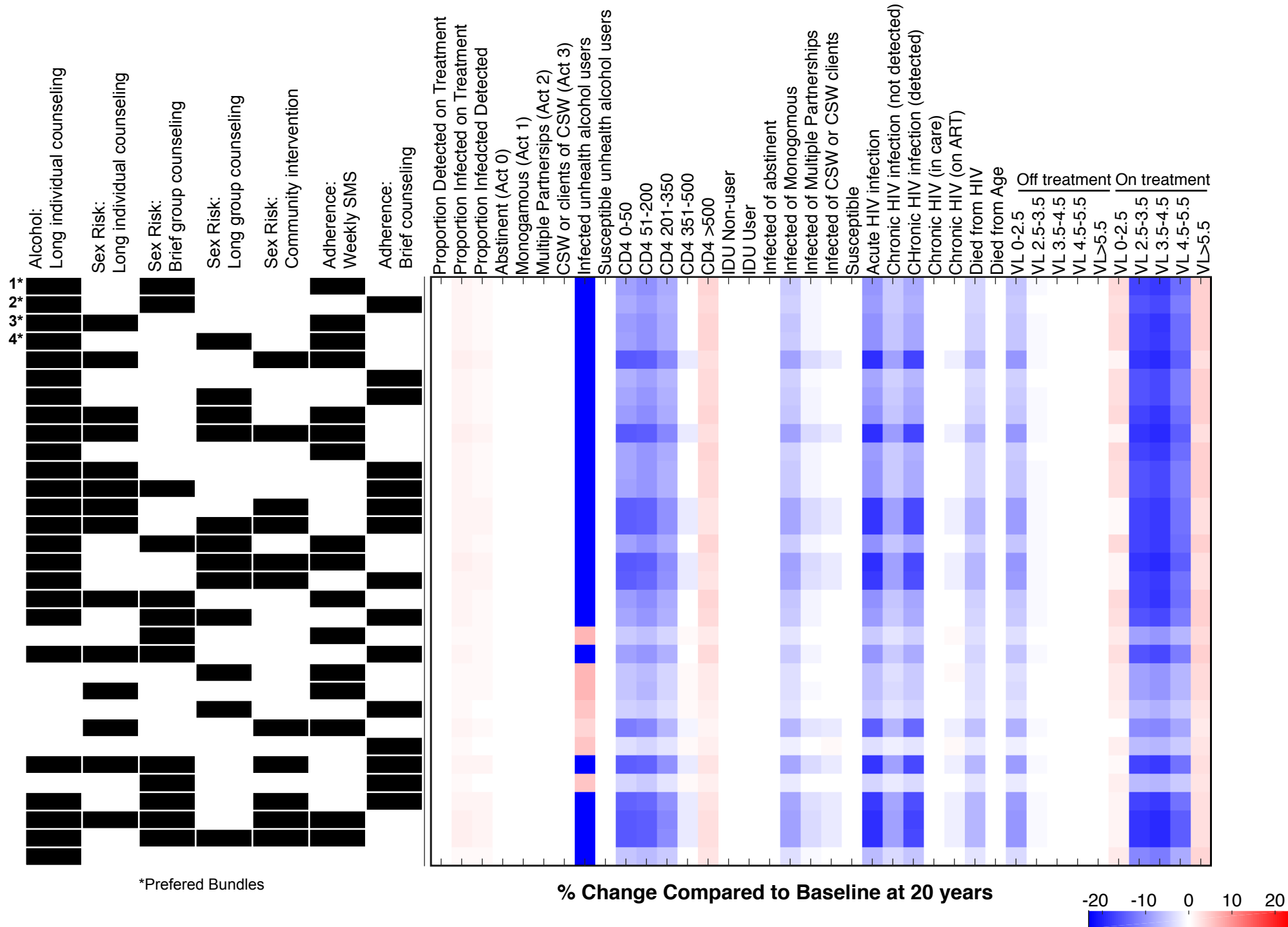

S4 Figure. Compartment dynamics in probabilistic intervention simulations

Supplement: S4 Fig — Mean % change in key model compartments at 20 years for top intervention bundles compared to baseline (no intervention) control across 1000 probabilistic runs. Color legends were split to allow for easier visualization. Color legend 1 corresponds to the first 3 variables and color legend 2 for the last 39. (PDF) [file pone.0184179.s004.pdf]
