# Supplementary material for: Betting on the fastest horse: Using computer simulation to design a combination HIV intervention for future projects in Maharashtra, India
Source: PLoS One. 2017 Sep 5;12(9):e0184179. doi: 10.1371/journal.pone.0184179 (PMC5584966; doi:10.1371/journal.pone.0184179)
Supplement: S3 Fig — a, Comparing model prevalence results with reported data for India. b, Comparing model incidence results with reported data for India. c, comparing annual proportion of people dying of HIV in model with reported data for India. d, comparing proportion of people with HIV on treatment compared with reported data for India. (PDF) [file pone.0184179.s003.pdf]

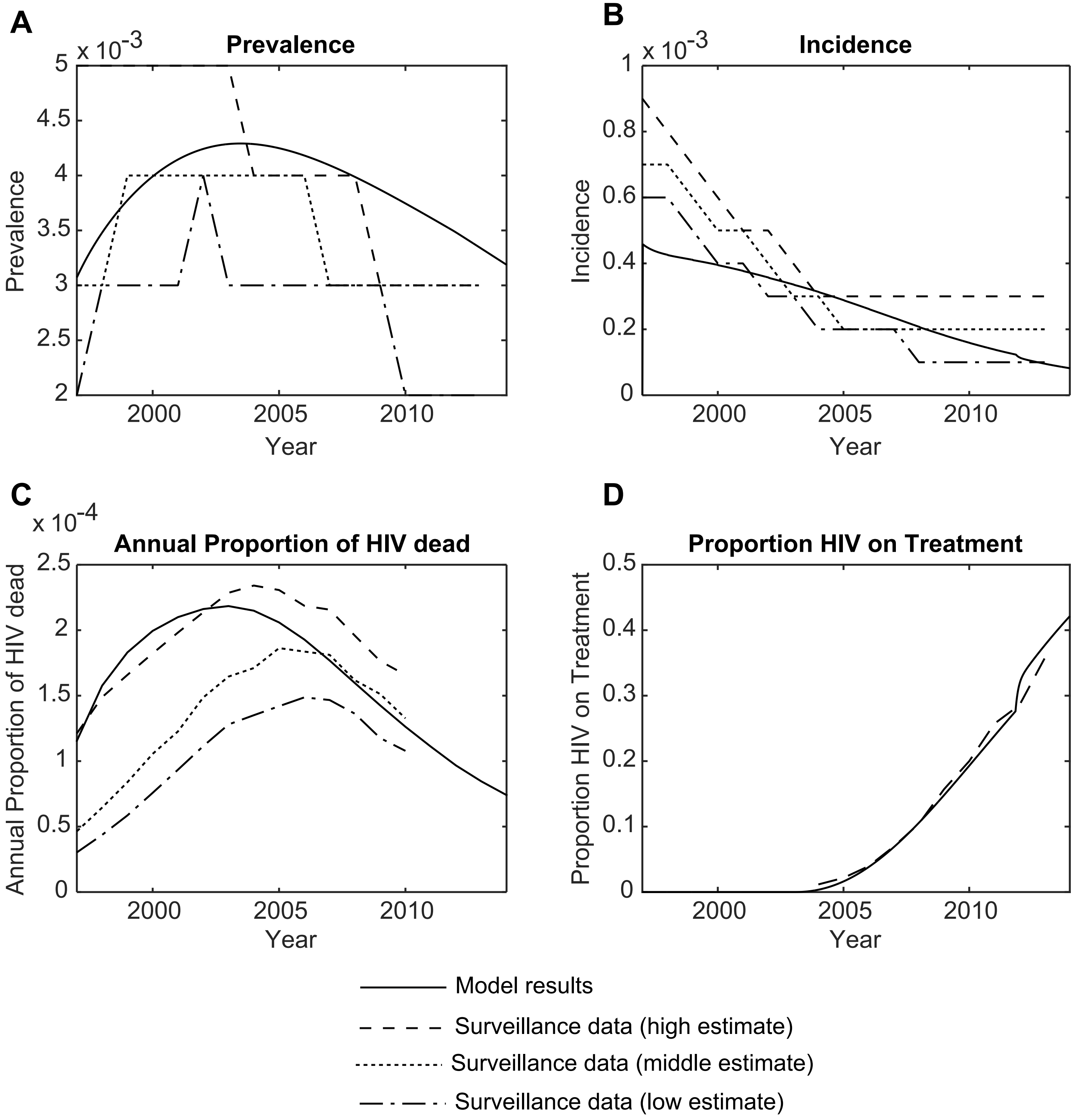

S3 Figure. Validation of HIV epidemic model. a, Comparing model prevalence results with reported data for India. b, Comparing model incidence results with reported data for India. c, comparing annual proportion of people dying of HIV in model with reported data for India. d, comparing proportion of people with HIV on treatment compared with reported data for India.
