# Supplementary material for: Betting on the fastest horse: Using computer simulation to design a combination HIV intervention for future projects in Maharashtra, India
Source: PLoS One. 2017 Sep 5;12(9):e0184179. doi: 10.1371/journal.pone.0184179 (PMC5584966; doi:10.1371/journal.pone.0184179)
Supplement: S5 Fig — (PDF) [file pone.0184179.s005.pdf]

S5 Figure. Histograms of probabilistic analysis for key input variables

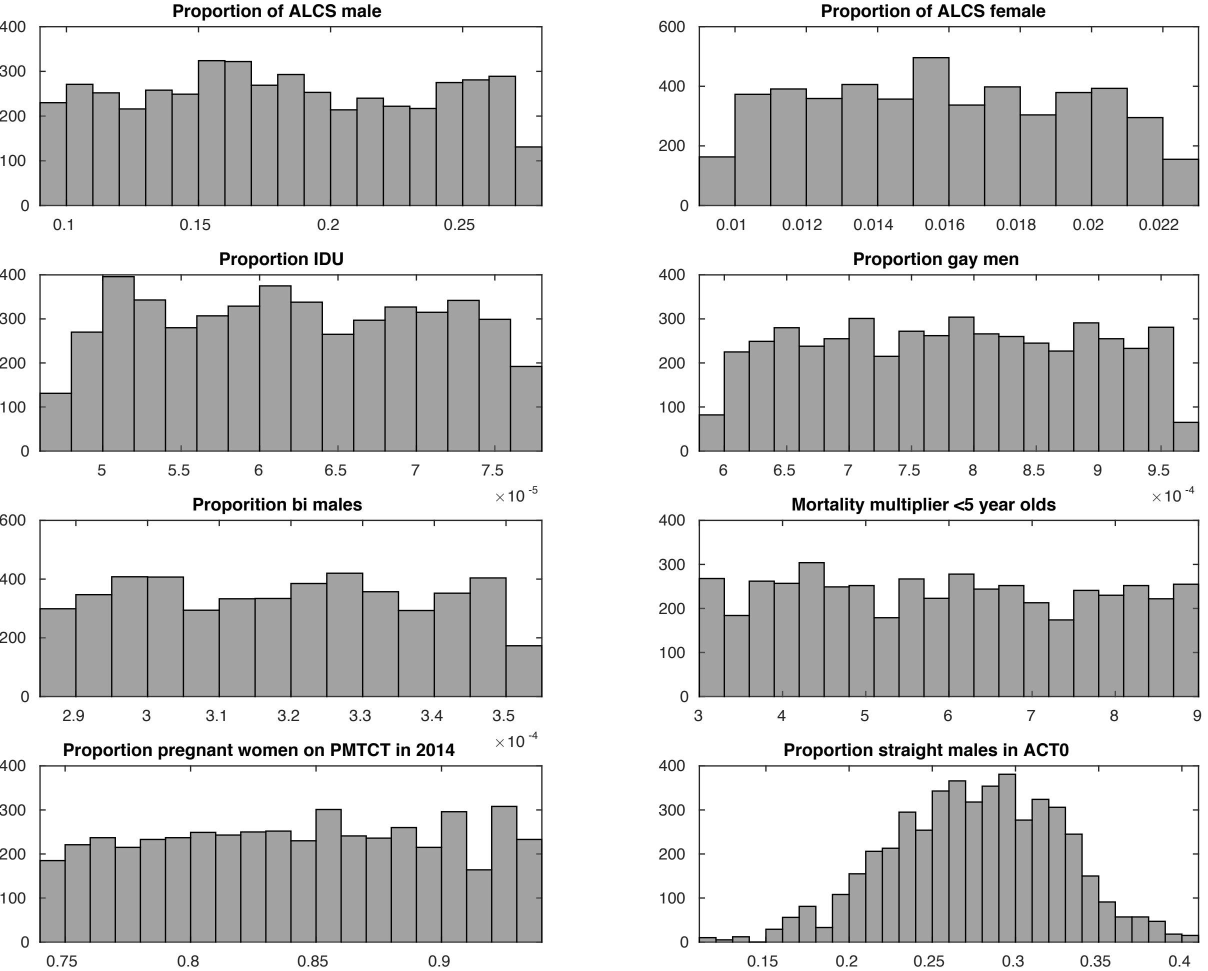

Proportion of ALCS male
0 100 200 300 400
0.1 0.15 0.2 0.25
Proportion of ALCS female
0 200 400 600
0.01 0.012 0.014 0.016 0.018 0.02 0.022
Proportion IDU
0 100 200 300 400
5 5.5 6 6.5 7 7.5
× 10^-5
Proportion gay men
0 100 200 300 400
6 6.5 7 7.5 8 8.5 9 9.5
× 10^-4
Proporition bi males
0 200 400 600
2.9 3 3.1 3.2 3.3 3.4 3.5
× 10^-4
Mortality multiplier <5 year olds
0 100 200 300 400
3 4 5 6 7 8 9
Proportion pregnant women on PMTCT in 2014
0 100 200 300 400
0.75 0.8 0.85 0.9
Proportion straight males in ACT0
0 100 200 300 400
0.15 0.2 0.25 0.3 0.35 0.4

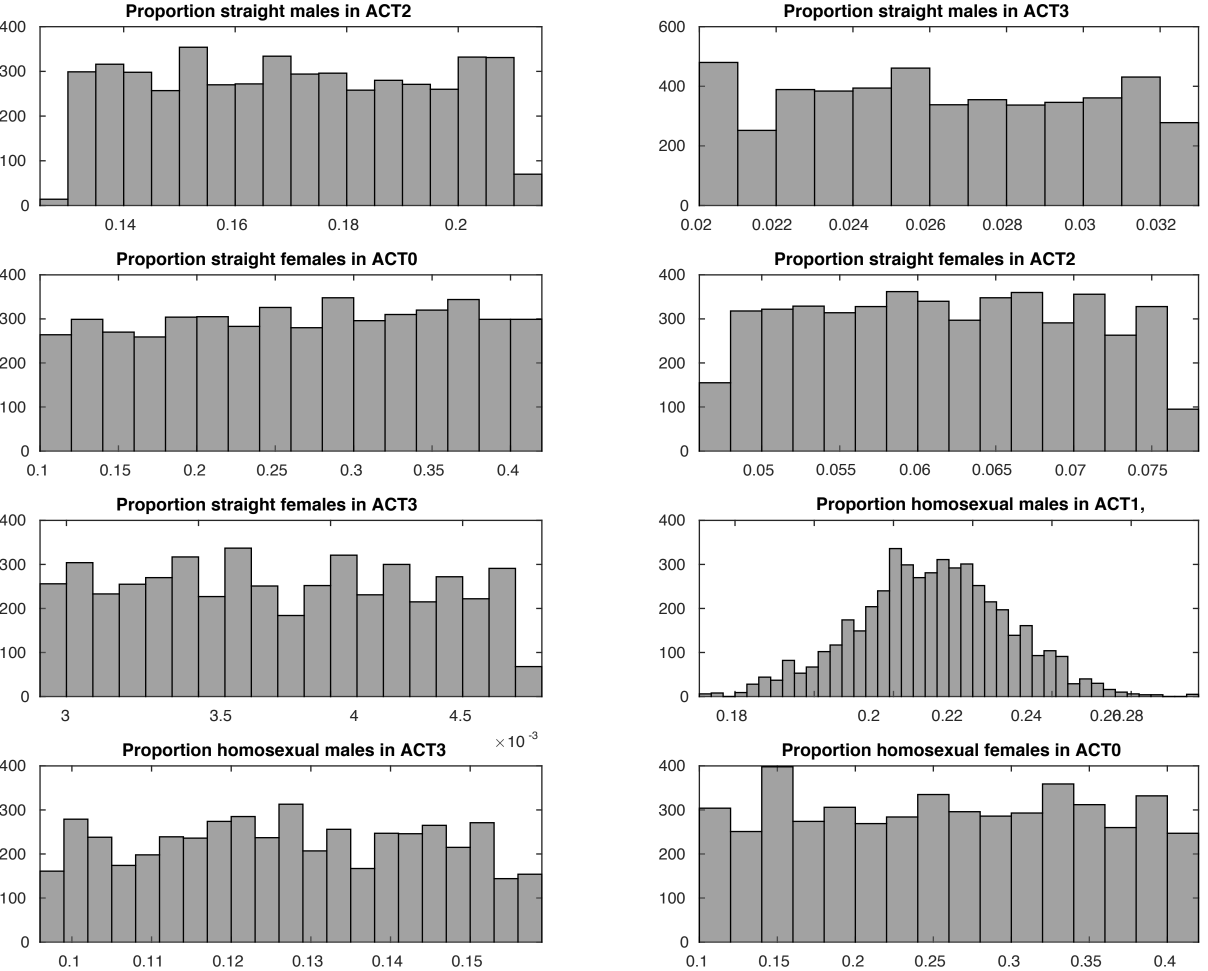
Proportion straight males in ACT2
0 100 200 300 400
0.14 0.16 0.18 0.2
Proportion straight males in ACT3
0 200 400 600
0.02 0.022 0.024 0.026 0.028 0.03 0.032
Proportion straight females in ACT0
0 100 200 300 400
0.1 0.15 0.2 0.25 0.3 0.35 0.4
Proportion straight females in ACT2
0 100 200 300 400
0.05 0.055 0.06 0.065 0.07 0.075
Proportion straight females in ACT3
0 100 200 300 400
3 3.5 4 4.5
×10^-3
Proportion homosexual males in ACT1,
0 100 200 300 400
0.18 0.2 0.22 0.24 0.26 0.28
Proportion homosexual males in ACT3
0 100 200 300 400
0.1 0.11 0.12 0.13 0.14 0.15
Proportion homosexual females in ACT0
0 100 200 300 400
0.1 0.15 0.2 0.25 0.3 0.35 0.4

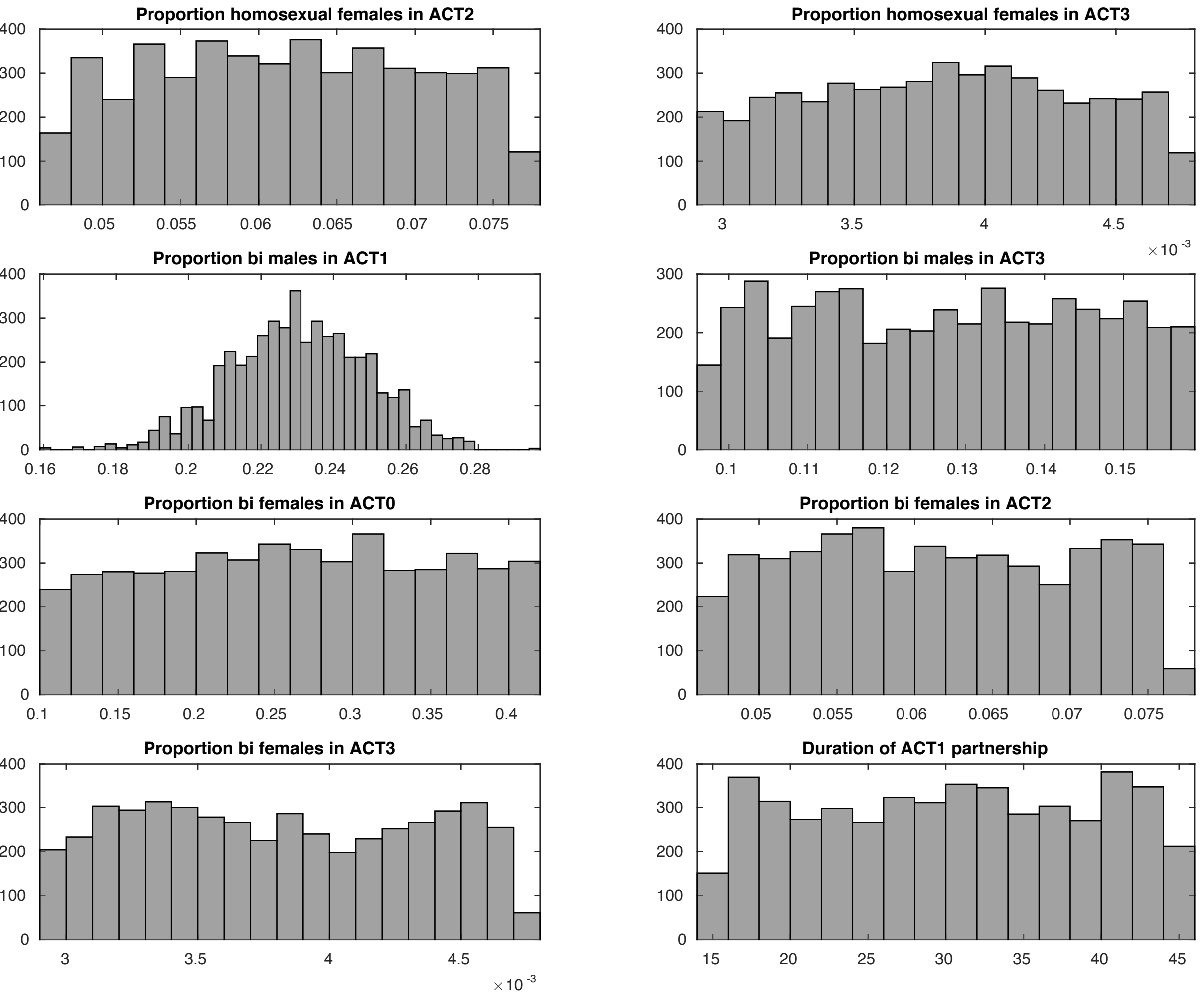
Proportion homosexual females in ACT2
400
300
200
100
0
0.05
0.055
0.06
0.065
0.07
0.075
Proportion homosexual females in ACT3
400
300
200
100
0
3
3.5
4
4.5
×10^-3
Proportion bi males in ACT1
400
300
200
100
0
0.16
0.18
0.2
0.22
0.24
0.26
0.28
Proportion bi males in ACT3
300
200
100
0
0.1
0.11
0.12
0.13
0.14
0.15
Proportion bi females in ACT0
400
300
200
100
0
0.1
0.15
0.2
0.25
0.3
0.35
0.4
Proportion bi females in ACT2
400
300
200
100
0
0.05
0.055
0.06
0.065
0.07
0.075
Proportion bi females in ACT3
400
300
200
100
0
3
3.5
4
4.5
×10^-3
Duration of ACT1 partnership
400
300
200
100
0
15
20
25
30
35
40
45

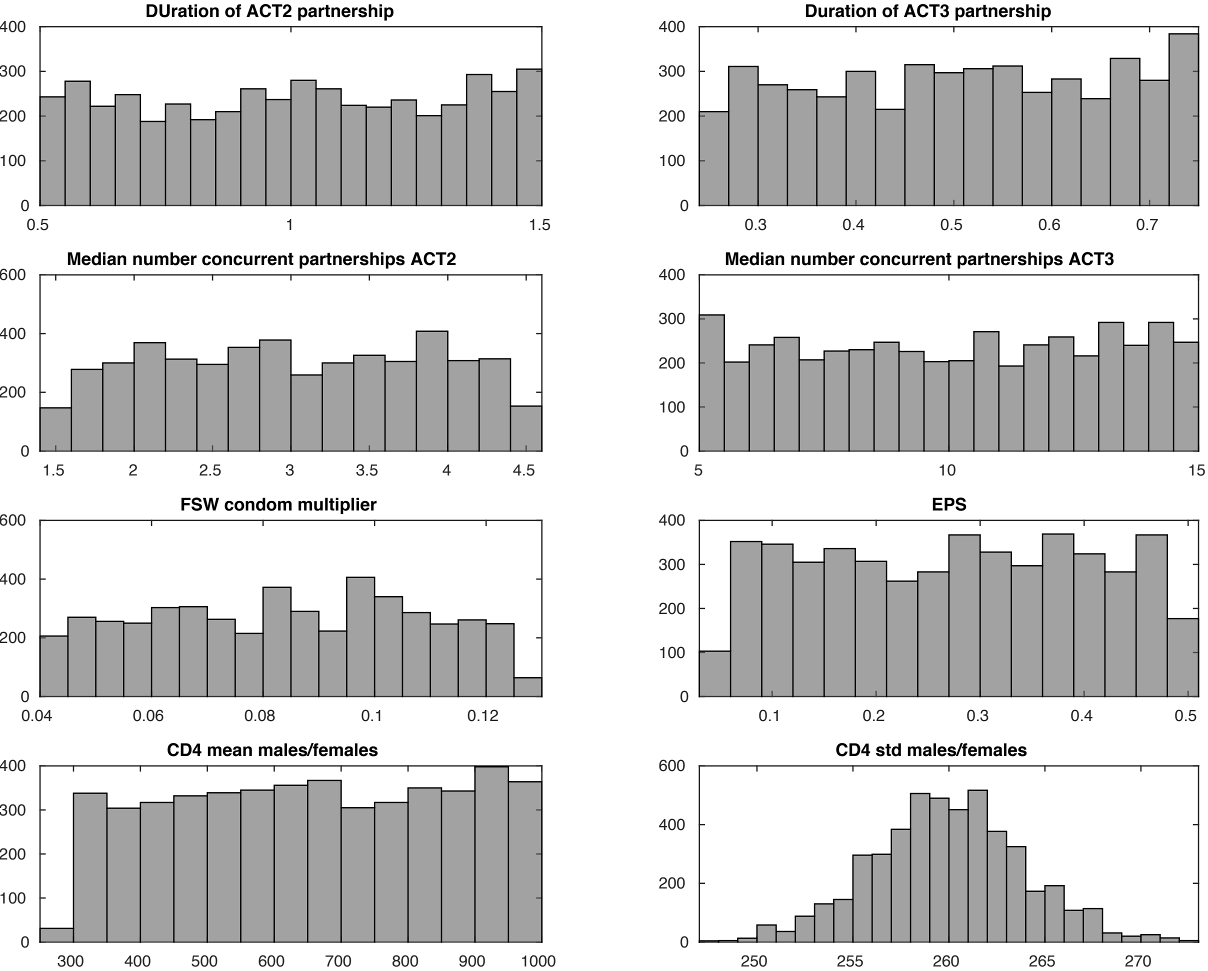
DUration of ACT2 partnership
400
300
200
100
0
0.5
1
1.5
Duration of ACT3 partnership
400
300
200
100
0
0.3
0.4
0.5
0.6
0.7
Median number concurrent partnerships ACT2
600
400
200
0
1.5
2
2.5
3
3.5
4
4.5
Median number concurrent partnerships ACT3
400
300
200
100
0
5
10
15
FSW condom multiplier
600
400
200
0
0.04
0.06
0.08
0.1
0.12
EPS
400
300
200
100
0
0.1
0.2
0.3
0.4
0.5
CD4 mean males/females
400
300
200
100
0
300
400
500
600
700
800
900
1000
CD4 std males/females
600
400
200
0
250
255
260
265
270

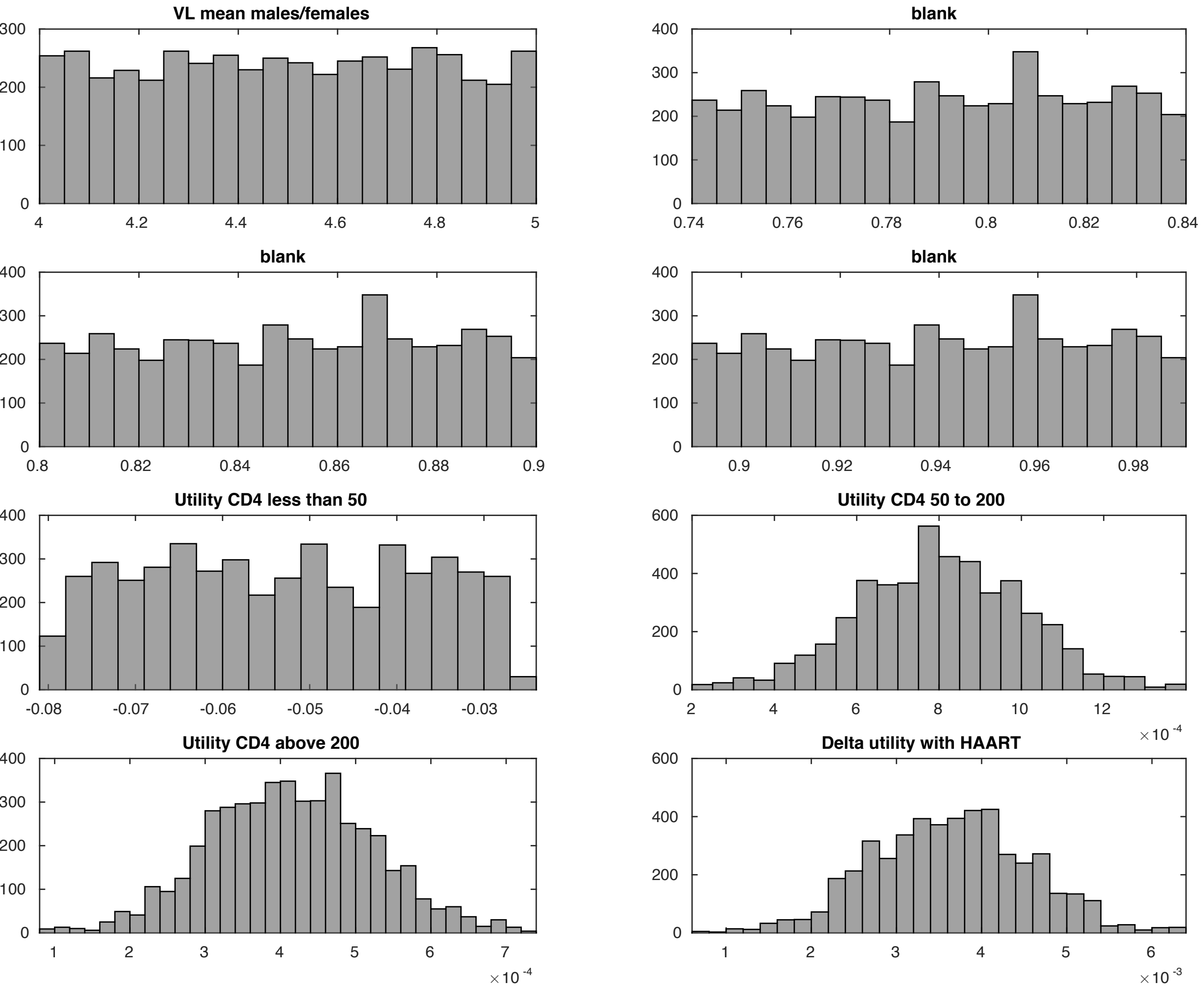
VL mean males/females
blank
blank
blank
Utility CD4 less than 50
Utility CD4 50 to 200
×10 -4
Utility CD4 above 200
×10 -4
Delta utility with HAART
×10 -3

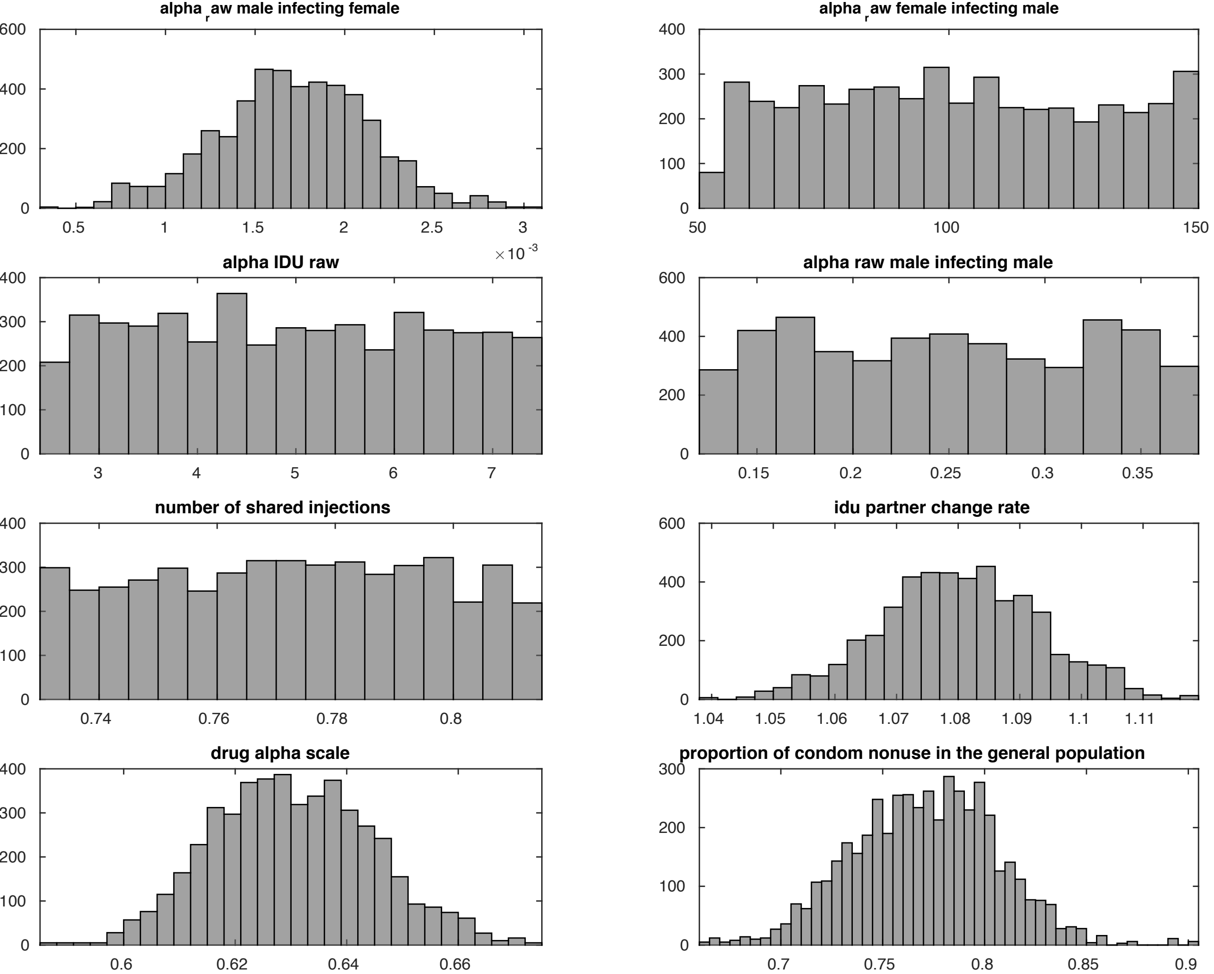
alpha raw male infecting female
0 200 400 600
0.5 1 1.5 2 2.5 3
×10^-3
alpha raw female infecting male
0 100 200 300 400
50 100 150
alpha IDU raw
0 100 200 300 400
3 4 5 6 7
alpha raw male infecting male
0 200 400 600
0.15 0.2 0.25 0.3 0.35
number of shared injections
0 100 200 300 400
0.74 0.76 0.78 0.8
idu partner change rate
0 200 400 600
1.04 1.05 1.06 1.07 1.08 1.09 1.1 1.11
drug alpha scale
0 100 200 300 400
0.6 0.62 0.64 0.66
proportion of condom nonuse in the general population
0 100 200 300
0.7 0.75 0.8 0.85 0.9

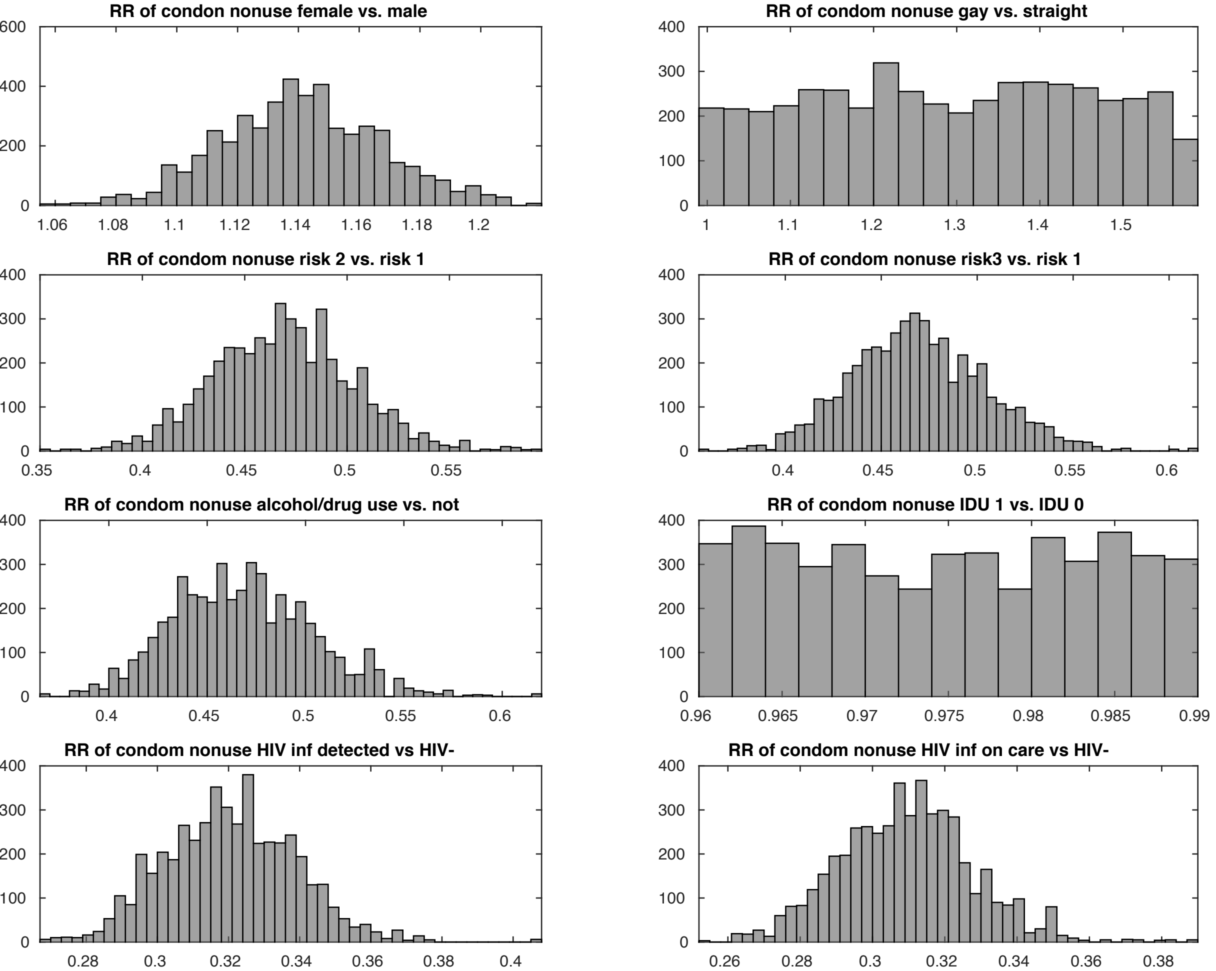

RR of condom nonuse female vs. male
600
400
200
0
1.06
1.08
1.1
1.12
1.14
1.16
1.18
1.2
RR of condom nonuse gay vs. straight
400
300
200
100
0
1
1.1
1.2
1.3
1.4
1.5
RR of condom nonuse risk 2 vs. risk 1
400
300
200
100
0
0.35
0.4
0.45
0.5
0.55
RR of condom nonuse risk3 vs. risk 1
400
300
200
100
0
0.4
0.45
0.5
0.55
0.6
RR of condom nonuse alcohol/drug use vs. not
400
300
200
100
0
0.4
0.45
0.5
0.55
0.6
RR of condom nonuse IDU 1 vs. IDU 0
400
300
200
100
0
0.96
0.965
0.97
0.975
0.98
0.985
0.99
RR of condom nonuse HIV inf detected vs HIV-
400
300
200
100
0
0.28
0.3
0.32
0.34
0.36
0.38
0.4
RR of condom nonuse HIV inf on care vs HIV-
400
300
200
100
0
0.26
0.28
0.3
0.32
0.34
0.36
0.38

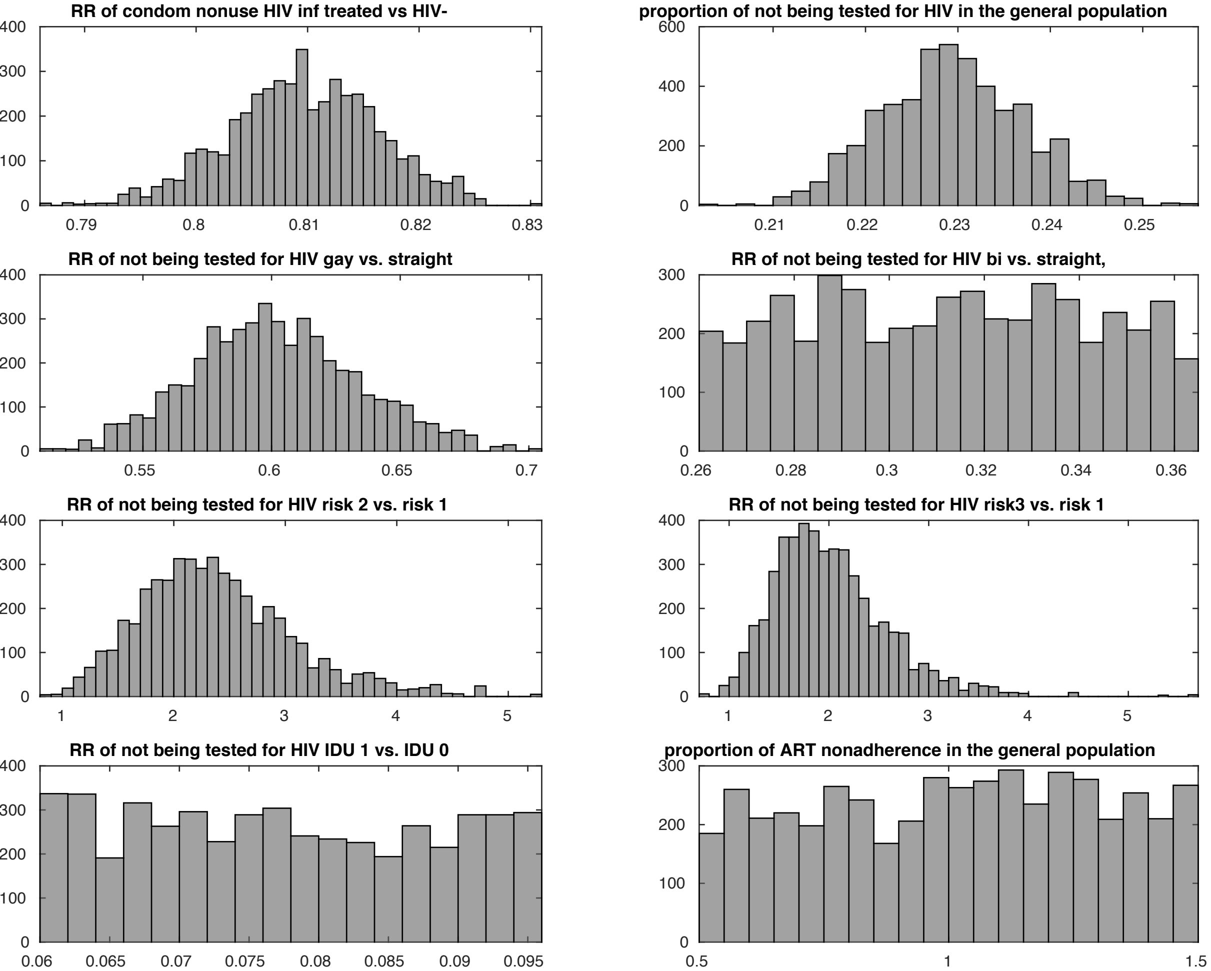
RR of condom nonuse HIV inf treated vs HIV-
0 100 200 300 400
0.79 0.8 0.81 0.82 0.83
proportion of not being tested for HIV in the general population
0 200 400 600
0.21 0.22 0.23 0.24 0.25
RR of not being tested for HIV gay vs. straight
0 100 200 300 400
0.55 0.6 0.65 0.7
RR of not being tested for HIV bi vs. straight,
0 100 200 300
0.26 0.28 0.3 0.32 0.34 0.36
RR of not being tested for HIV risk 2 vs. risk 1
0 100 200 300 400
1 2 3 4 5
RR of not being tested for HIV risk3 vs. risk 1
0 100 200 300 400
1 2 3 4 5
RR of not being tested for HIV IDU 1 vs. IDU 0
0 100 200 300 400
0.06 0.065 0.07 0.075 0.08 0.085 0.09 0.095
proportion of ART nonadherence in the general population
0 100 200 300
0.5 1 1.5

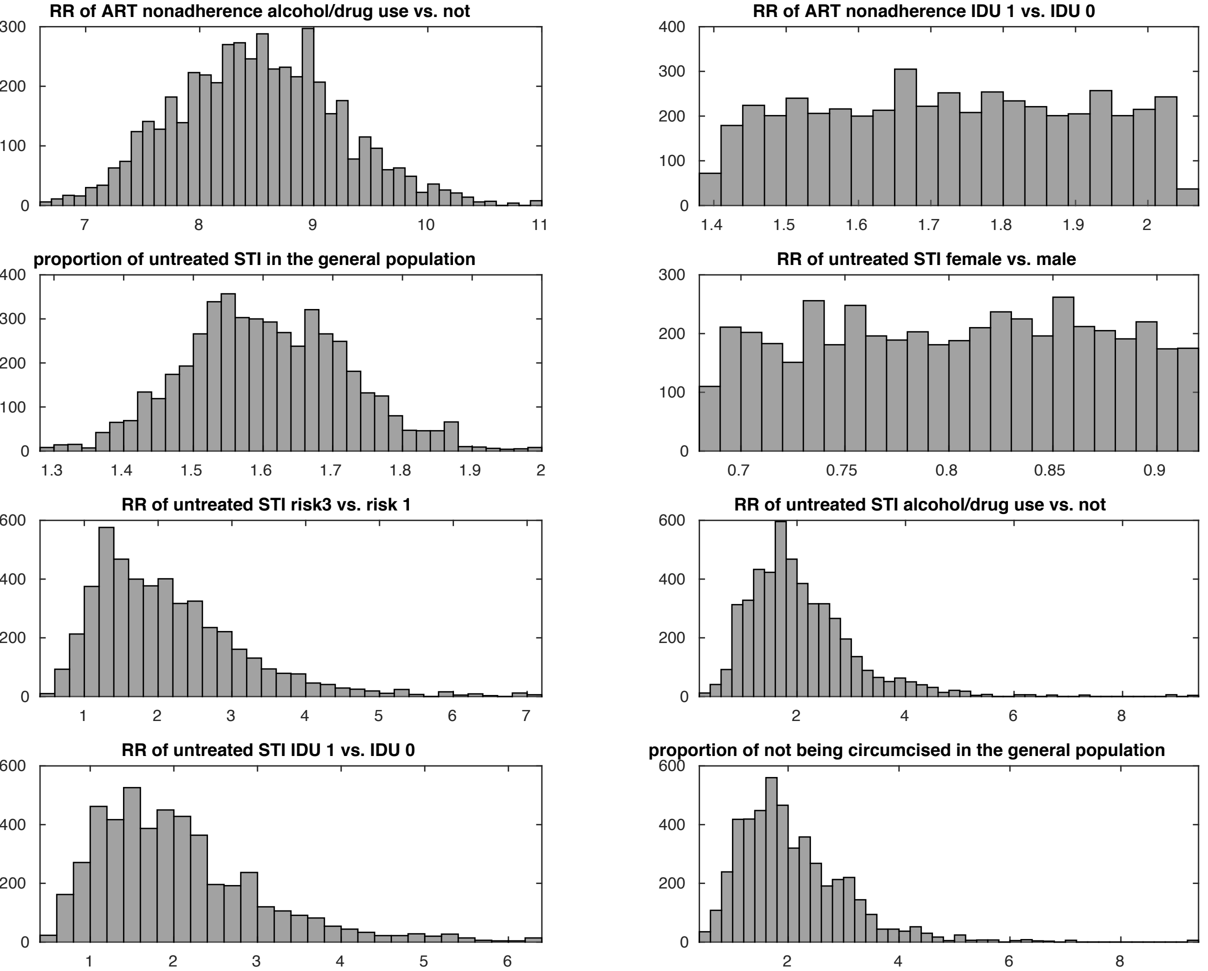
RR of ART nonadherence alcohol/drug use vs. not
0 100 200 300
7 8 9 10 11
RR of ART nonadherence IDU 1 vs. IDU 0
0 100 200 300 400
1.4 1.5 1.6 1.7 1.8 1.9 2
proportion of untreated STI in the general population
0 100 200 300 400
1.3 1.4 1.5 1.6 1.7 1.8 1.9 2
RR of untreated STI female vs. male
0 100 200 300
0.7 0.75 0.8 0.85 0.9
RR of untreated STI risk3 vs. risk 1
0 200 400 600
1 2 3 4 5 6 7
RR of untreated STI alcohol/drug use vs. not
0 200 400 600
2 4 6 8
RR of untreated STI IDU 1 vs. IDU 0
0 200 400 600
1 2 3 4 5 6
proportion of not being circumcised in the general population
0 200 400 600
2 4 6 8

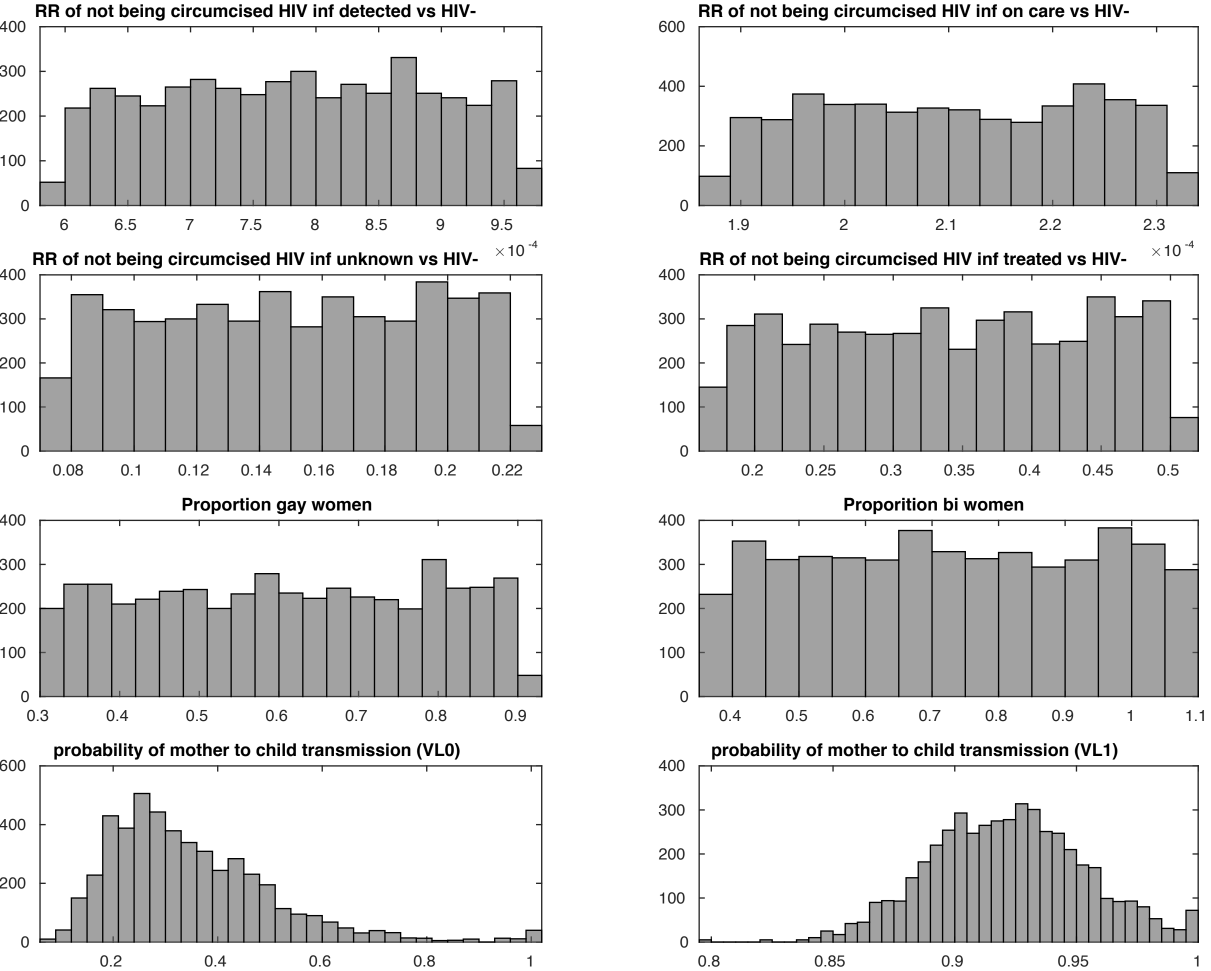

RR of not being circumcised HIV inf detected vs HIV-
×10^-4
RR of not being circumcised HIV inf on care vs HIV-
×10^-4
RR of not being circumcised HIV inf unknown vs HIV-
RR of not being circumcised HIV inf treated vs HIV-
Proportion gay women
Proporition bi women
probability of mother to child transmission (VL0)
probability of mother to child transmission (VL1)

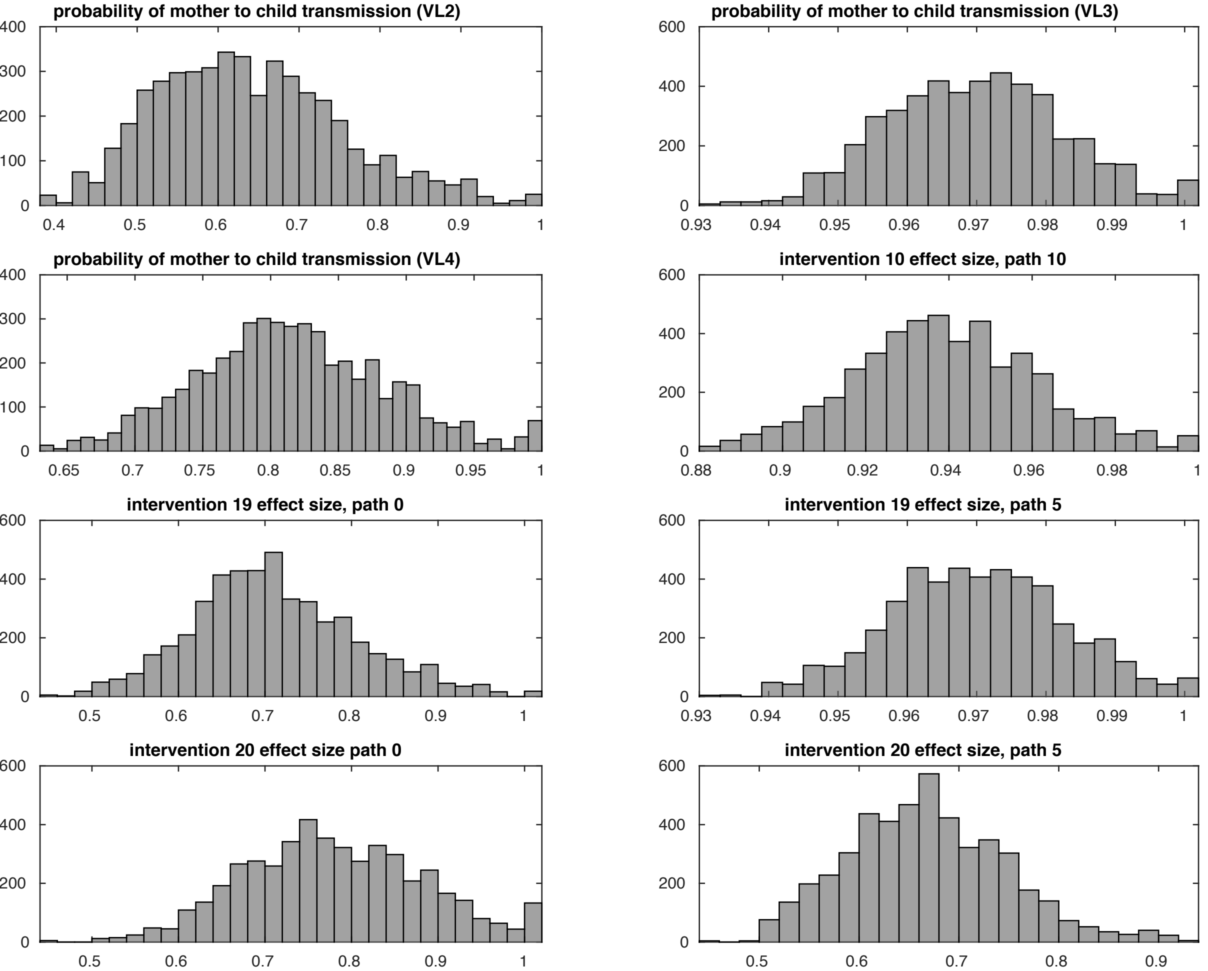
probability of mother to child transmission (VL2)
0.4 0.5 0.6 0.7 0.8 0.9 1
probability of mother to child transmission (VL3)
0.93 0.94 0.95 0.96 0.97 0.98 0.99 1
probability of mother to child transmission (VL4)
0.65 0.7 0.75 0.8 0.85 0.9 0.95 1
intervention 10 effect size, path 10
0.88 0.9 0.92 0.94 0.96 0.98 1
intervention 19 effect size, path 0
0.5 0.6 0.7 0.8 0.9 1
intervention 19 effect size, path 5
0.93 0.94 0.95 0.96 0.97 0.98 0.99 1
intervention 20 effect size path 0
0.5 0.6 0.7 0.8 0.9 1
intervention 20 effect size, path 5
0.5 0.6 0.7 0.8 0.9

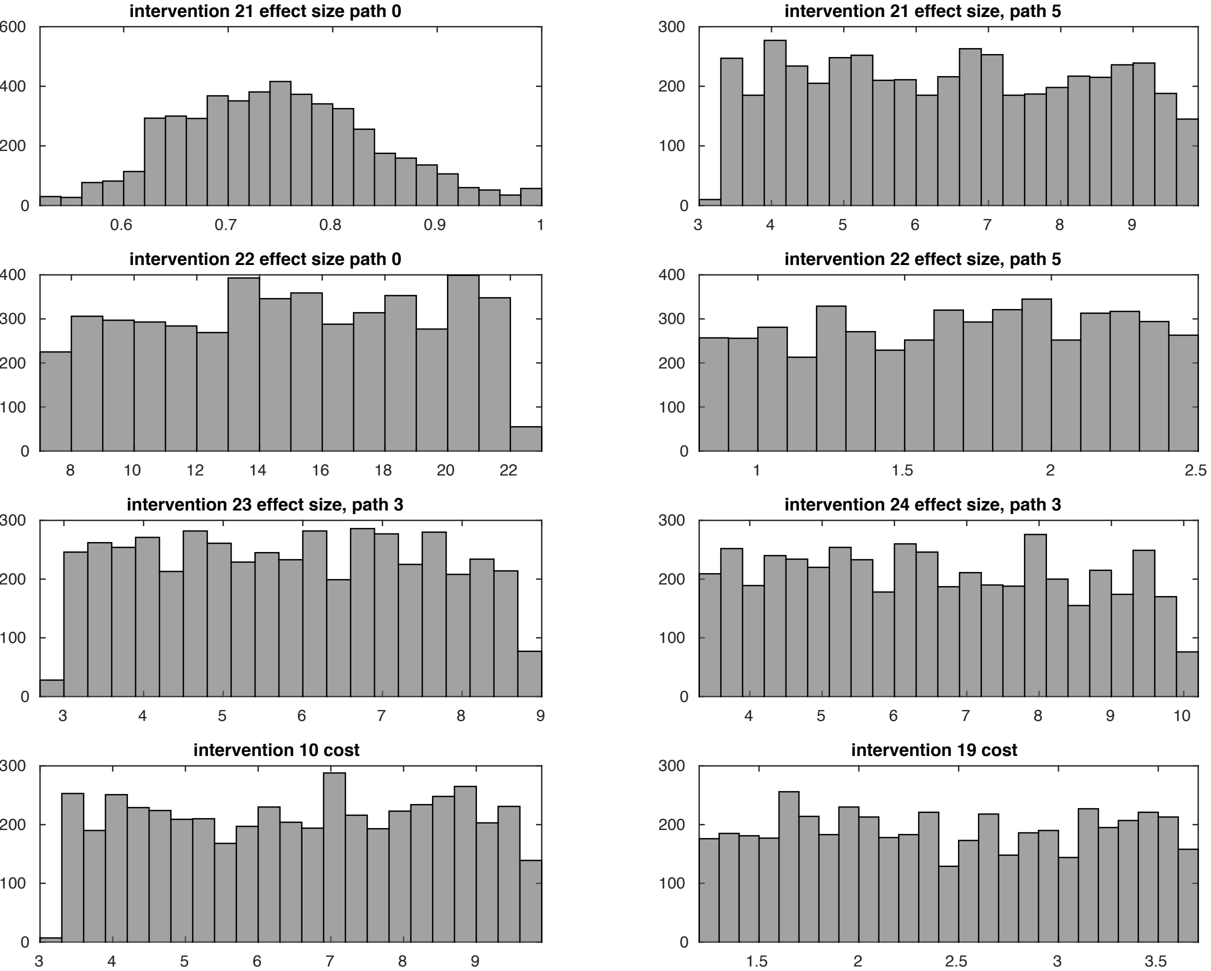
intervention 21 effect size path 0
intervention 21 effect size, path 5
intervention 22 effect size path 0
intervention 22 effect size, path 5
intervention 23 effect size, path 3
intervention 24 effect size, path 3
intervention 10 cost
intervention 19 cost
