# Supplementary material for: Betting on the fastest horse: Using computer simulation to design a combination HIV intervention for future projects in Maharashtra, India
Source: PLoS One. 2017 Sep 5;12(9):e0184179. doi: 10.1371/journal.pone.0184179 (PMC5584966; doi:10.1371/journal.pone.0184179)
Supplement: S6 Fig — a, Graphical representation of intervention bundles identified on the efficient frontier across 100 probabilistic runs of all combinations of 7 interventions. b, focused for lower end of discounted cost (0.888–0.893 Billion USD). c, intervention bundle details corresponding to a and b. (PDF) [file pone.0184179.s006.pdf]

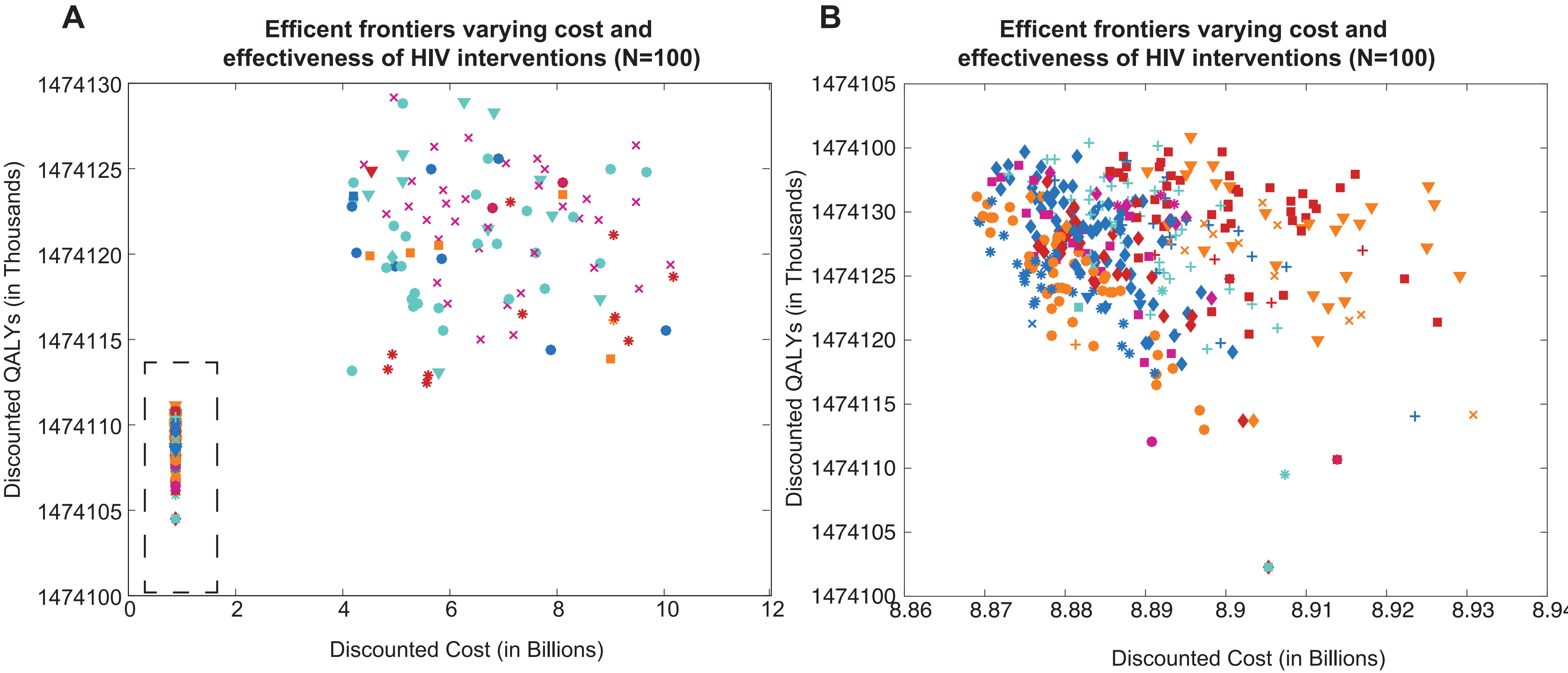

S6 Figure. Intervention cost and effectiveness probabilistic analysis. a, Graphical representation of intervention bundles identified on the efficient frontier across 100 probabilistic runs of all combinations of 7 interventions. b, focused for lower end of discounted cost (0.888-0.893 Billion USD). c, intervention bundle details corresponding to a and b.

Bundle #	Marker	Intervention on Frontier							% Times on frontier
		Alcohol: Long individual counseling	Adherence: Weekly SMS	Adherence: Brief counseling	Sex Risk : Long individual counseling	Sex risk: Brief group counseing	Sex Risk: Long group counseling	Sex risk: Community intervention	
1	◆	X	X			X			63
2	●	X		X		X			47
3	■	X	X		X				46
4	+	X	X				X		44
5	×	X	X		X			X	38
6	✳	X		X					29
7	▼	X	X		X		X		27
8	◆	X		X			X		27
9	●	X	X		X		X	X	26
10	■	X	X						22
11	+	X		X	X				19
12	×	X		X	X		X		12
13	✳	X		X	X		X	X	10
14	▼	X		X	X			X	10
15	◆	X	X			X	X		9
16	●	X	X				X	X	8
17	■	X		X			X	X	5
18	+	X	X		X	X			4
19	✳	X		X	X	X			3
20	◆	X		X		X	X		3
21	▼		X			X			3
22	×		X				X		3
23	●		X		X			X	2
24	+		X		X				2
25	■			X			X		2
26	▼	X	X		X	X		X	1
27	✳	X	X			X	X	X	1
28	■	X		X	X	X		X	1
29	◆	X		X		X		X	1
30	×	X							1
31	●			X		X			1
32	+			X					1

Figure S6. Continued
