# Supplementary material for: Betting on the fastest horse: Using computer simulation to design a combination HIV intervention for future projects in Maharashtra, India
Source: PLoS One. 2017 Sep 5;12(9):e0184179. doi: 10.1371/journal.pone.0184179 (PMC5584966; doi:10.1371/journal.pone.0184179)
Supplement: S7 Fig — a., Discounted cost of care and treatment (2014 USD), b., total discounted QALYs for all and c., HIV infected and HIV infected in treatment, d., the number of new infections and HIV infected deaths over 20 years and e., the mean number of new infections and HIV deaths per infected per year. (PDF) [file pone.0184179.s007.pdf]

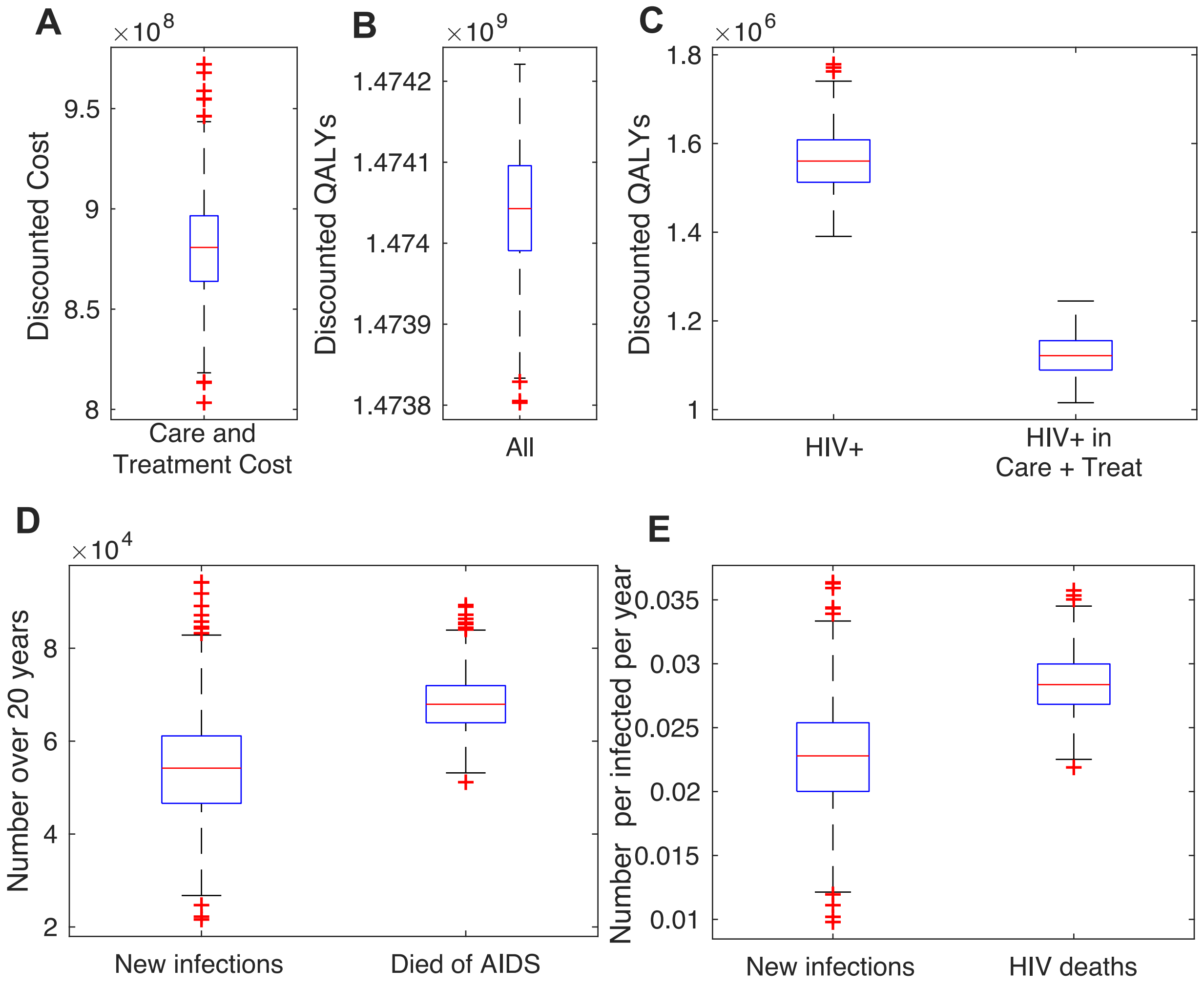

S7 Figure. Distribution of epidemic outcomes across 1000 probabilistic baseline runs after 20 year simulations.
