# Supplementary material for: Betting on the fastest horse: Using computer simulation to design a combination HIV intervention for future projects in Maharashtra, India
Source: PLoS One. 2017 Sep 5;12(9):e0184179. doi: 10.1371/journal.pone.0184179 (PMC5584966; doi:10.1371/journal.pone.0184179)
Supplement: S1 Table — (PDF) [file pone.0184179.s008.pdf]

S1 Table. Key model inputs

Sexual Risk Characteristics	Base Case	Probabilistic Range	Distribution	Ref.
Age of Sexual Debut	19	17-21	Uniform	[29]
Proportion of men who are homosexual	0.06%	0.06%-0.1%	Uniform	[28]
Proportion of men who are bisexual	0.04%	0.029%-0.035%	Normal	[28]
Proportion of women who are homosexual	0.06%	---	---	Assumption
Proportion of women who are bisexual	0.04%	---	---	Assumption
Proportion of straight males who are abstinent	27.75%	21%-41%	Normal	[32]
Proportion of straight males who are in stable, monogamous relationships	57.33%	--	--	[32]
Proportion of straight males in multiple, concurrent relationships (Class 3)	12.9%	12.9%-21.2%	Normal	[32]
Proportion of straight males in multiple, concurrent relationships (Class 4)	2.0%	2%-3.3%	Uniform	[30]
Proportion of bisexual males who are abstinent	0%	---	---	--
Proportion of bisexual males who are in stable, monogamous relationships	23.0%	15.41%-30.35%	Normal	[28]
Proportion of bisexual males in multiple, concurrent relationships (Class 3)	67.5%	---	---	--
Proportion of bisexual males in multiple, concurrent relationships (Class 4)	9.5%	7.44%-11.98%	Normal	[28]
Proportion of homosexual males who are abstinent	0%	---	---	--
Proportion of homosexual males who are in stable, monogamous relationships	23.0%	15.41%-30.35%	Normal	[28]
Proportion of homosexual males in multiple, concurrent relationships (Class 3)	67.5%	---	---	--
Proportion of homosexual males in multiple, concurrent relationships (Class 4)	9.5%	7.44%-11.98%	Normal	[28]
Proportion of straight females who are abstinent	26.0%	12.27%-43.78%	Normal	[29]
Proportion of straight females who are in stable, monogamous relationships	69.0%	53.45%-84.78%	Normal	[29]
Proportion of straight females in multiple, concurrent relationships (Class 3)	4.7%	1.99%-2.73%	Normal	[29]
Proportion of straight females in multiple, concurrent relationships (Class 4)	0.3%	0.3%-3%	Uniform	[30]
Proportion of bisexual females who are abstinent	26.0%	12.27%-43.78%	Normal	[29]
Proportion of bisexual females who are in stable, monogamous relationships	69.0%	53.45%-84.78%	Normal	[29]
Proportion of bisexual females in multiple, concurrent relationships (Class 3)	4.7%	1.99%-2.73%	Normal	[29]
Proportion of bisexual females in multiple, concurrent relationships (Class 4)	0.3%	0.3%-3%	Uniform	[30]
Proportion of homosexual females who are abstinent	26.0%	12.27%-43.78%	Normal	[29]
Proportion of homosexual females who are in stable, monogamous relationships	69.0%	53.45%-84.78%	Normal	[29]
Proportion of homosexual females in multiple, concurrent relationships (Class 3)	4.7%	1.99%-2.73%	Normal	[29]
Proportion of homosexual females in multiple, concurrent relationships (Class 4)	0.3%	0.3%-3%	Uniform	[30]

S1 Table. Key model inputs (continued)

HIV Transmission	Base Case	Sensitivity Range	Distribution	Ref.
**Sexual Transmission**				
Transmission risk per sex act (F→M)	0.00042	0.5x-1.5x	normal	60
Transmission risk per sex act (M→F)	0.00081	0.5x-1.5x	normal	60
Transmission risk per sex act (M→M)	0.00169	0.5x-1.5x	normal	60
Relative risk of transmission if VL category 0-2.5 log copies/ml	0.16	--	--	62
Relative risk of transmission if VL category 2.5-3.5 log copies/ml	1.87	--	--	62
Relative risk of transmission if VL category 3.5-4.5 log copies/ml	6.54	--	--	62
Relative risk of transmission if VL category 4.5-5.5 log copies/ml	8.85	--	--	62
Relative risk of transmission if VL category >5.5 log copies/ml	9.03	--	--	62
Degree of assortative mixing between age and sexual activity classes (0=assortative, 1=proportionate)	0.20	0.05-0.50	uniform	56
Average duration (years) of stable, monogamous partnerships	30.0	0.5x-1.5x	uniform	2
Average duration (years) of partnership in activity group 2	1.0	0.5x-1.5x	uniform	2
Average duration (years) of partnership in activity group 3	0.5	0.5x-1.5x	uniform	2
Median number of concurrent partnerships for activity group 1	1.0	--	--	55
Median number of concurrent partnerships for activity group 2	3.0	0.5x-1.5x	uniform	55
Median number of concurrent partnerships for activity group 3	10.0	0.5x-1.5x	uniform	55
Relative risk reduction of HIV seroconversion when using condoms	0.80	--	--	65
Relative risk reduction of HIV seroconversion if treated STI	0.40	--	--	66
Relative risk reduction of HIV seroconversion if circumcised	0.59	--	--	67
**Injection Drug Use**				
Transmission risk per injection	0.0036	0.5x-1.5x	normal	61
Proportion of population that uses IV drugs	4.99E-5	0.0018-0.0054	uniform	28
Proportion of IDU with unsafe injection practices	32%	--	--	Assumption
Number of needle sharing partners per year	5	0.5x-1.5x	uniform	63
Shared injections per year	102	54-150	Uniform	61

S1 Table. Key model inputs (continued)

Biological/behavioral modifiers of transmission	Base Case	Sensitivity Range	Distribution	Ref.
**General Population Risk**				
Prevalence of untreated STI	0.06	0.06-0.10	Normal	[59]
Probability of not being circumcised	0.80	0.68-0.92	Uniform	[68]
Prevalence of condom nonuse most or all of the time	0.73	0.73-0.82	Normal	[29]
Probability/rate of not being tested for HIV	0.98	0.947-0.999	Uniform	[31]
Probability of linkage to care once HIV diagnosed	0.30	---	---	
Prevalence of ART non-adherence	0.26	0.26-0.36	Normal	[58]
Probability of loss to follow-up	0.26	0.23-0.29	Normal	[69]
**Female vs. Male**	**RR**			
RR of condom non-use	1.08	1.06 – 1.11	Log-Normal	[70]
**Homosexual vs. Straight**	**RR**			
RR of condom non-use	0.63	0.6 – 0.66	Log-Normal	[71]
RR of not being tested for HIV	0.31	0.28 – 0.35	Log-Normal	[71]
**Bisexual vs Straight**	**RR**			
RR of not being tested for HIV	0.31	0.28 – 0.35	Log-Normal	Assumption
**Non-monogamous vs. monogamous**	**RR**			
RR of condom non-use	1.14	1.13 – 1.16	Log-Normal	[71]
RR of not being tested for HIV	0.81	0.79 – 0.82	Log-Normal	[71]
**High risk (class 4) vs. monoagmous**	**RR**			
RR of condom non-use	0.16	0.15 – 0.18	Log-Normal	[71]
RR of not being tested for HIV	0.23	0.22 – 0.25	Log-Normal	[71]
RR of untreated STI	8.85	7.4 – 10.3	Log-Normal	[70]
**Alcohol use vs. non alcohol use**	**RR**			
RR of condom non-use	1.29	1 – 1.58	Uniform	[36]
RR of ART non-adherence	2.33	1.17 – 3.5	Uniform	[38,39,41]
RR of untreated STI	1.72	1.4 – 2.05	Uniform	[43,72,73]
**IDU vs. non-IDU**	**RR**			
RR of condom non-use	0.62	0.55 – 0.7	Log-Normal	[71]
RR of not being tested for HIV	0.6	0.54 – 0.67	Log-Normal	[71]
RR of ART non-adherence	2	1 – 3	Normal	Assumption
RR of untreated STI	1.43	1.22 – 1.63	Log-Normal	[71]
RR of LTFU prior to linkage	2	1 – 3	Normal	Assumption
**HIV+ vs. HIV-**	**RR**			
RR of condom non-use	0.47	0.4 – 0.54	Log-Normal	[74]
RR of not being circumcised	2.22	1.16 – 6.67	Log-Normal	[75]

S1 Table. Key model inputs (continued)

HIV disease related demographics	Base Case	Sensitivity Range	Distribution	Ref.
Mean CD4 count (SD) for newly infected individuals	644 (260)	294-994 (65-585)	Uniform (Normal)	[33]
Mean log viral load (SD) for newly infected females	4.46 (0.99)	4-5	Uniform	[33]
CD4 count threshold for treatment initiation	200	---	---	[76]
Fertility rate (range, depending on age)	0.0027-0.23	---	---	[77]
**Costs (2014 USD)**				
1st line ART monthly costs	$11.86	--	--	[47]
2nd line ART monthly costs	$49.27	--	--	[47]
Annual routine costs if in HIV care and treatment program	$132.18	--	--	[49]
Annual inpatient hospitalization costs if patient has AIDS	$347.25	--	--	[49]
HIV-1 viral load test	$49.54	--	--	[47]
CD4+ count test	$6.32	--	--	[47]
Initial proportion alcohol abuse problems, males	0.185	0.0925-0.2775	Normal	[57]
Initial proportion alcohol abuse problems, females	0.020	0.0096-0.0224	Normal	Assumption
