# Supplementary material for: Betting on the fastest horse: Using computer simulation to design a combination HIV intervention for future projects in Maharashtra, India
Source: PLoS One. 2017 Sep 5;12(9):e0184179. doi: 10.1371/journal.pone.0184179 (PMC5584966; doi:10.1371/journal.pone.0184179)
Supplement: S1 File — (DOCX) [file pone.0184179.s009.docx]

**S1 File. Technical Appendix**

1. **HIV Epidemic Model**

We modified a previously described compartmental, deterministic, SIR-type model originally built to describe heterosexual transmission of HIV within an East African setting.[1,2] Our new model has been modified to represent heterosexual, homosexual and injection drug use (IDU) transmission of HIV in Maharashtra, India.

For additional information or for a working copy of the C++ code used to conduct the analyses outlined in this manuscript please contact the director of our mathematical modeling team at Kimberly.Nucifora@nyumc.org.

**1.2 Epidemic model population definition**

The simulated population was divided into ten 5-yr age groups (*i*), two genders (*k*), three sexual orientations (o) and four sexual activity classes (*l*) (**Table S1**). Additionally, the population was divided based on alcohol use into those who are healthy alcohol users versus those with unhealthy alcohol use and injection drug use into those who participate in injection drug use and those who do not. Additional details on these compartments can be found in **section 4**. These risk groups were selected to capture differences in HIV prevalence, population size, sexual behaviors and transmission risk. For those persons living with HIV/AIDS their disease was represented with a CD4 count category (*cd*), and a viral load (VL) category (*v*). In addition, the spectrum of infection and engagement in care was represented in a status category (*y*) (Table S1). The specific combination of age, gender, sexual activity class may be (where appropriate) referred to using “risk strata” (subscript *p)* throughout the remainder of this document. The combination of CD4 and viral load may be referred to as “HIV state” (subscript *h*) throughout the remainder of this document.

**Table S1.** Components of population matrix

| **Population parameter** | **Abbreviation/Subscript** |  | **Subgroups** |
| --- | --- | --- | --- |
| Age | *i* | *p* | 0-4, 5-9, 10-14, 15-19, 20-24, 25-29, 30-34, 35-39, 40-45, 45-49 |
| Gender | *k* |  | Women(*k=1*), Men (*k=2*) |
| Sexual orientation | *o* |  | Straight (*o=1*), Gay (*o=2*), Bisexual (*o=3*) |
| Sexual activity | *l* |  | Abstinent (*l=1*), Monogamous (*l=2*), Multiple partnerships (*l=3*), CSW or clients of CSW (*l=4)* |
| Unhealthy alcohol use | *a* |  | Non-user/healthy user (a=0), Untreated unhealthy alcohol user (a=1), Treated and “cured” unhealthy alcohol user (a=2), Treated and “not cured” unhealthy alcohol user (a=3) |
| Injection drug use (IDU) | *idu* |  | Non-injection drug user (*idu=0)*, Injection drug user (*idu=1*) |
| CD4 count | *cd* | *h* | 0-50, 51-200, 201-350, 351-500, >500 cells/mm^3^ |
| VL | *v* |  | 0-2.5, 2.5-3.5, 3.5-4.5, 4.5-5.5, >5.5 log copies/ml |
| Status | *y* | | Susceptible (y=1), Acute HIV infection (y=2), Chronic HIV infection (not detected) (y=3), Chronic HIV (detected) (y=4),  Chronic HIV (in care) (y=5), Chronic HIV (on ART) (y=6) |

**1.3 Epidemic model structure and equations**

The compartmental model was created through a system of nonlinear differential equations for each spectrum of care group (referred to as “status” in **Table S1**) further subdivided by age (a), CD4 category (cd), VL category (v), and risk group (p). Across the spectrum of care, transition from susceptible (S_r_) to acute HIV infection (I^1^_r_) occurs as a result of new transmission events; transition from I^1^_r_ to undetected, chronic HIV infection (I^2^_r_ ) occurs at a constant rate and represents the natural history of acute HIV infection; transition from I^2^_r_ to chronic HIV, detected (I^3^_r_) occurs as a result of HIV testing and this rate can vary in relation to changes in this probability. Transition from I^3^_r_ to chronic HIV, in care (I^4^_r_) occurs as a result of linkage to care. Finally, transition from I^4^_r_ to HIV, on ART (I^5^_r_) occurs once those in care initiate ART, which is directly related to the assumed ART eligibility criteria. From I^5^_r_ the only transition that can be made is to death (**Figure S1**). As discussed below, HIV progression (i.e. transitions between CD4 and VL compartments) and HIV-related mortality was modeled using rates developed from a stochastic state-transition model [[1](#_ENREF_1), [2](#_ENREF_2)].


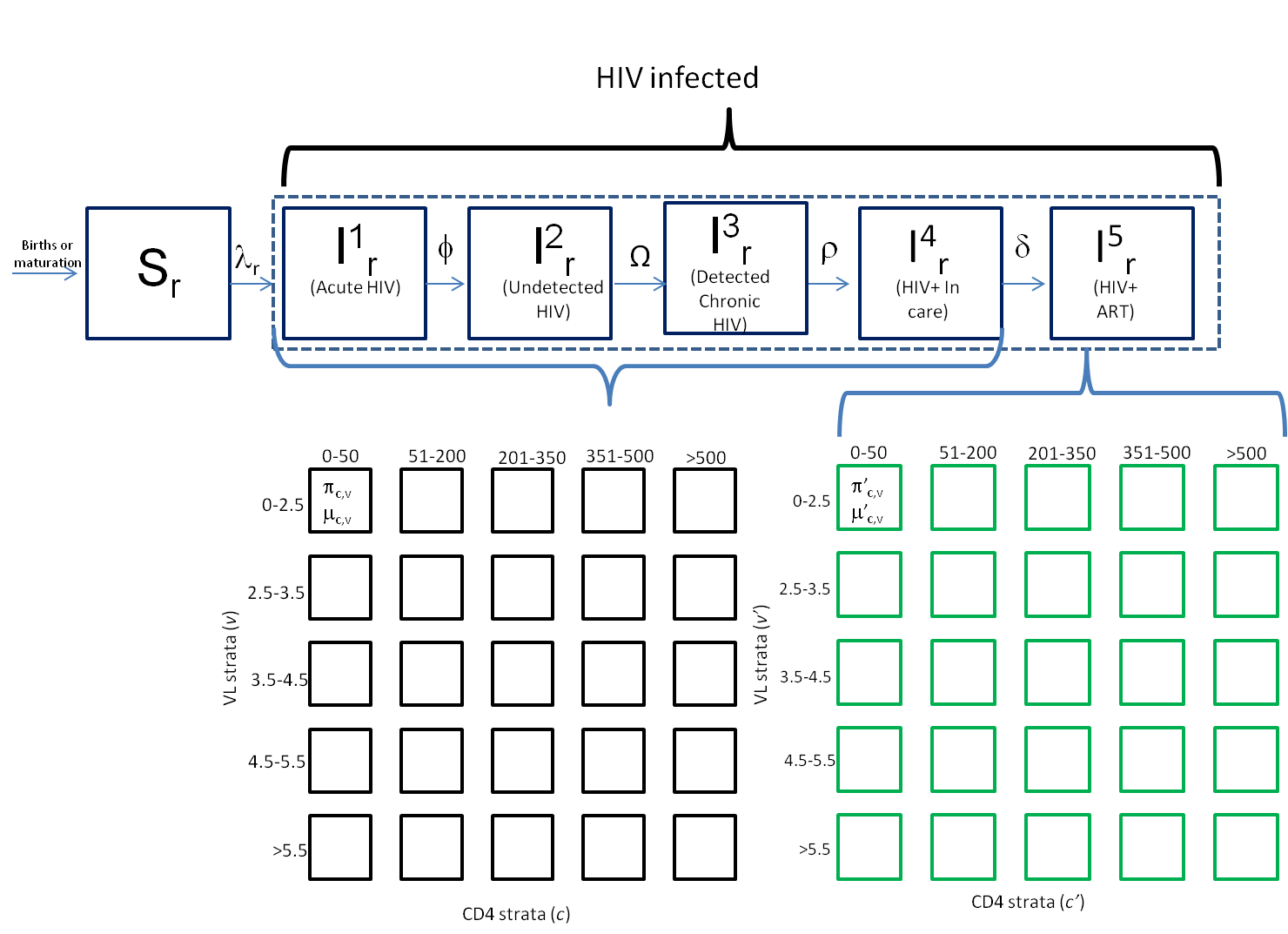


HIV disease progression model

(equilibrium rate tables)

HIV transmission model

**Figure S1.** Schematic diagram of HIV transmission model

**1.3.1 Equations governing transitions**

N = Total population

N’ = HIV infected population

S = N-N’ = Susceptible (HIV-) population

p = risk strata- given combination of age strata (a), gender (k), sexual activity class (l)

h= disease strata – given combination of CD4 strata (cd) , VL strata (vl) and HIV resistance (m) category

μ_h_= HIV-related mortality rate (for a given CD4 strata (cd), VL strata (v), and resistance (m) category)

μ’_h_= HIV-related mortality rate if on cART

μ_age_ = age – related mortality rate

λ = force of infection

γ = birth or maturation

π = rate of transition between any two CD4, VL strata, and resistance categories

φ = flow from acute to chronic HIV infection

η = rate of transition from acute to chronic HIV infection

Ω = rate of HIV testing [= - ln(1-P_test_)/t)]

ρ = rate of linkage to care [= - ln(1-P_link_)/t)]

δ = rate of initiation of ART (once in care)

P_test_= probability/rate of annual HIV testing

P_LTC_= probability/rate of linkage to care (if detected)

P_ART_= probability/rate of initiation of ART (if in care)

1. *d*S_r_/*d*t _=_ γ _r_ – λ^t^_r_ (*t*)S_r_ – μ_age_
2. *d*I^1^_r_/*d*t = λ^t^_r_ (*t*)S_r_ – φ_r_ - μ_age_ - μ*_cd,v_* (where φ_r_ = η*N’_r_)
3. *d*I^2^_r_  / *d*t = φ_r_ - Ω - μ_age_ - μ*_cd,v_*
4. *d*I^3^_r_  / *d*t = Ω – ρ - μ_age_ - μ*_cd,v_*
5. *d*I^4^_r_/*d*t = ρ_r_ – δ_r_ - μ_age_ - μ*_cd,v_* (where δ_r_ = N’_r_*π*_cd<threshold,v_* * PART)
6. *d*I^5^/*d*t = δ_r_ - μ_age_ - μ’*_cd,v_*

**1.4 HIV transmission**

The population is described by two genders (male and female), three sexual orientation groups and four sexual activity groups. Each activity group is defined by a desired relationship duration in years *d* and desired number of concurrent partners *r* . The mathematical framework builds on published work by Garnett & Anderson.[3,4] Following their structure, the population defined by the model is stratified by gender *k*, age *i*, sexual orientation *o*, and sexual activity class *l*. Individuals form partnerships with gender *k'*  and sexual orientation *o’* from age class *i’* and activity class *l’*. The notation for duration d_kolik’o’l’i’_ would refer to the duration of a partnership formed between someone of sex *k*, activity group *l* and age group *i* with someone of sex k’ from activity group *l’*, age group *i’*. In addition to the above parameters, we set a desired frequency of sexual acts (number of acts per year) which applies to both genders and a probability of transmission per sexual act. The following steps were undertaken to create and balance the transmission simulation.

1. **Who Can Partner With Who matrix**

The potential for mixing between individuals of one gender-orientation combination with individuals of another gender-orientation combination are defined by the Who Can Partner With Who (WCPWW) matrix. The matrix is defined as follows:

WCPWW_kolik’o’l’i’_

= 1 if a=straight, k≠k’, o’=straight or bi

= 1 if a=homosexual, k=k’, o’=homosexual or bi

= 1 if o=bi, k=k’, o’=homosexual or bi

= 1 if o=bi, k=k’, o’=straight or bi

= 0 otherwise

**b) Proportional mixing matrix incorporating concurrency**

We begin by calculating the proportionate mixing matrix*.* This indicates the probability that an individual in a given group will partner with an individual from each other group under random, proportionate mixing. Note that instead of using partner change rate in this calculation, we use concurrency to determine the number of partnerships available at a given moment in time.

The proportionate mixing matrix ρ^p^ is defined by:

$\rho_{kolik^{'}o^{'}l^{'}i^{'}}^{p}= \frac{N_{k'o'l'i'}r_{k'o'l'i'}{WCPWW}_{kolik'o'l'i'}}{\sum_{k^{'}=1}^{n_{1}} \sum_{o^{'}=1}^{n_{2}} \sum_{l^{'}=1}^{n_{3}} \sum_{i^{'}=1}^{n_{4}} N_{k'o'l'i'}r_{k'o'l'i'}{WCPWW}_{kolik'o'l'i'}}$

(1)

**c) The fully assortative mixing matrix.**

The assortative mixing matrix partners persons of one age and activity level with persons of the same age and activity level.

The assortative mixing matrix ρ^a^ is defined by:

$$\rho_{kolik^{'}o^{'}l^{'}i^{'}}^{a}= \frac{N_{k'o'l'i'}r_{k'o'l'i'}{WCPWW}_{kolik'o'l'i'}\delta_{ii'll'}}{\sum_{k^{'}=1}^{n_{1}} \sum_{o^{'}=1}^{n_{2}} \sum_{l^{'}=1}^{n_{3}} \sum_{i^{'}=1}^{n_{4}} N_{k'o'l'i'}r_{k'o'l'i'}{WCPWW}_{kolik'o'l'i'}}$$

(2)

**d) Specifying level of assortativeness.**

The level of assortativeness (preference of an individual to mix with someone of the same age and activity group) is denoted by ε (eq 2). Here, d_ii’_ is the Kronecker delta (i.e. d_ii’_ = 1 if i = i’ and 0 otherwise). When $\varepsilon$ = 0, mixing is entirely assortative and when $\varepsilon$ =1 mixing is proportionate. These methods are described by Garnett and Anderson [3,4].

The final mixing matrix incorporating ε is:

$$\rho_{kolik^{'}o^{'}l^{'}i^{'}}=\varepsilon\rho_{kolik^{'}o^{'}l^{'}i^{'}}^{p}+(1-\varepsilon)\rho_{kolik^{'}o^{'}l^{'}i^{'}}^{a}$$

(3)

**d) Duration**

When a man and woman form a partnership, we make the assumption that the partner wanting the shorter relationship controls the relationship duration. Thus the duration of the partnership is the minimum of each partner's desired duration.

d_kolik’o’l’i’_ = min(d_koli_, d_k’o’l’i’_)

**e) Number of partnerships offered at any moment in time**

The number of partnerships offered by a group “koli” to a partner group “k'o’l’i’” is the product of the number in that group, the number of concurrent partners for that group, and the probability that the partner will be from the partner group.

numPartnerships_kolik’o’l’i’_ = N_koli_ * r_koli_ * ρ_kolik’o’l’i’_

**f) Compromise on number of partnerships**

When two groups in a partnership are offering each other unequal numbers of partnerships, the lower number of partnerships prevails. Thus one group may get fewer partnerships than it desired.

numPartnershipsCompromise_kolik’o’l’i’_ = numPartnershipsCompromise_k’o’l’i’koli_ = min(numPartnerships_kolik’o’l’i’_, numPartnerships_k’o’l’i’koli_)

**g) Compromise on concurrency**

Now that one group may have had to compromise on the number of partnerships it can obtain with a partner group, we have to revise the concurrency for that group, specific to that partnership. Revisiting our equation from step 4 but using the number of partnerships after compromising,

numPartnershipsCompromise_kolik’o’l’i’_ = N_koli_ * r_koli_ * ρ_kolik’o’l’i’_

Solving for concurrency,

r_kolik’o’l’i’_ = numPartnershipsCompromise_kolik’o’l’i’_ / (N_koli_ * ρ_kolik’o’l’i’_)

**h) Frequency of sexual acts**

In our model, we assume a overall desired frequency *F* for all individuals. This overall desired frequency is independent of the number of concurrent partners. Thus an individual with only one partner with have sex acts of frequency F with that one partner. An individual with *r* concurrent partners will have frequency F/r with each partner. In step 6, we showed that because one group may have had to compromise their number of partnerships, their concurrency with a partner group likewise had to be adjusted. We calculate actual frequency f_klmij_ for a group with a specific partner group as

f_kolik’o’l’i’_ = F / r_kolik’o’l’i’_

**i) Compromise on frequency**

Two individuals who form a partnership must each experience the same frequency within the partnership. If their calculated desired frequencies with each other are unequal, one partner will have to compromise on their desired frequency. In this model, we assume that the lower desired frequency prevails. Thus,

f_kolik’o’l’i’_ = f_k’o’l’i’koli_ = min(f_kolik’o’l’i’_, f_k’o’l’i’koli_)

**j) Acts per Partnership**

The number of acts within the course of a partnership is equal to the frequency of sex acts multiplied by the duration.

acts_kolik’o’l’i’_ = f_kolik’o’l’i’_ * d_kolik’o’l’i’_

**k) Partner change rate**

The partner change rate c is equal to the number of partnerships per year (in series) multiplied by the number of concurrent partnerships r. The number of partnerships in series is the inverse of the duration. Thus,

c_kolik’o’l’i’_ = 1/d_kolik’o’l’i’_ * r_kolik’o’l’i’_ = r_kolik’o’l’i’_/d_kolik’o’l’i’_

**l) Transmission probability (beta) per partnership**

The transmission probability per partnership is calculated by

β = 1 - (1- α) ^acts^

where α is the transmission probability per sex act.

**m) Force of Infection**

The force of infection (l) is defined by

$$\lambda_{koli}\left( t \right)=\sum_{k^{'}=1}^{n1} \sum_{o^{'}=1}^{n2} \sum_{i^{'}=1}^{n3} \sum_{l^{'}=1}^{n4} c_{kolik'o'l'i'}(t)\rho_{kolik'o'l'i'}\frac{\sum_{s=1}^{n3} \beta_{sko}Y_{{sk}^{'}o^{'}l^{'}i^{'}}(t)}{N_{k'o'l'i'}(t)}$$

(4)

where s refers to the infectious states and Ysk'mj(t) is the number of sexual partners of age group j and activity group m in that infectious state. The term Y_sk’o’l’I’_(t)/N_k’o’l’I’_(t) is then the proportion of sexual partners who are infected.

1.4.1 Calculation of probability of transmission per act and per partnership

We refer to the transmission probability per partnership as β and the transmission probability per sexual act as α Alpha(α) constants were set based on literature reviews and vary by type of partnership and the infecting partner [Table S4]. These constants can be modified by HIV disease status (acute vs. chronic infection), viral load, adherence and specific interventions which act or promote different behavioral or biological modifiers that increase or decrease this probability (e.g. condom use during sex act will decrease the probability of transmission).

Therefore, under different conditions α is calculated using the following equations:

*Infected partner not receiving treatment (and no alpha modifying interventions):*

α_final_= α* α^m^_v_ (6)

where α^m^_v_ is is the alpha modifier for a viral load strata *v*

To account for the higher VL (and likely transmission probability resulting) associated with the acute HIV state, if the partner of a susceptible person was in an acute HIV state (I^1^_r_) then the viral load of the infected person was conservatively estimated to be 1.5 log units greater than its set point (and a different VL modifier (α^m^_v_) applied to the above equation [[5](#_ENREF_5)].

*Infected partner receiving treatment (and no alpha modifying interventions):*

First, an “on treatment” viral load strata for infected population is determined as follows:

VL_treat_ = VL - (VLdec * P_adh_)

and then

VL_treat_ is then assigned a new VL strata (*v’ )* category

α^mt^_v_  is the alpha modifier of the new VL strata on treatment *v’*

The final alpha value is then calculated as in equation (6).

To calculate β we assumed a binomial process where the number of trials referred to the average number of sex acts per partnership (Acts) and the probability of successful transmission is described by α_kKV_ as above. Therefore, beta was calculated using the following equation.

β = 1 -(1 - α_final_)^acts^ (7)

1. **HIV disease progression**

**2.1 Disease progression model calibration**. Based on a previously calibrated disease progression (stochastic, Markov state transition model) module for East Africa [1,2], we developed a model of HIV progression specific to Maharashtra, India using data from AMPATH (*www.ampath.com*). Our pre-specified calibration criteria involved survival (**Figure S2A**), time to first line ART treatment failure (**Figure S2B**), and CD4 response to ART treatment (**Figure S2C**).

We used statistical methods, previously published by our group, to adjust passively assessed patient mortality by employing random sampling of patients lost to follow-up with active surveillance and estimating overall mortality by combining the data with patients who remained in follow-up. In addition, we estimated the longitudinal trajectories of CD4 counts. Unfortunately, the later data are potentially biased in unpredictable ways (for example, tracking median CD4 count over time might overestimate the true CD4 trajectory as patients with lower CD4 counts may die or be lost-to-follow-up at higher rates than patients with higher CD4 counts). For this reason, we placed the greatest emphasis on ensuring that the survival curves, produced by our simulation model, fit the clinical data well.

**Figure S2**. Calibration of HIV progression model. A. Comparing model generated survival curve with administrative data from India. B. Comparing model generated time to treatment failure with reported data for India. C. Comparing model generated CD4 recovery after ART initiation to administrative data from India.

**2.2 Generation of equilibrium results from disease progression model for use in epidemic, compartmental model**

HIV disease progression was modeled by the inclusion of stationary rates developed from a previously developed and validated computer simulation [[1](#_ENREF_1)]. {Braithwaite, 2005 #190}Within the epidemic compartmental model CD4^+^ count was categorized into five mutually exclusive compartments (*cd*) [0-50, 50-200, 200-350, 350-500, >500 cells/mm^3^. Viral load was categorized into five strata (*v*) [0-2.5, 2.5-3.5, 3.5-4.5, 4.5-5.5, >5.5 log copies/ml]. Upon initialization, compartments representing PLWHA at the start of the simulation were assigned a CD4 count, and VL as represented from the distribution in **Table S2**. This distribution describing the CD4 and VLs for PLWHA at the start of the simulation were created using published data.[5,6]

All persons infected with HIV after the start of the simulation were assigned a CD4 strata of >500 cells/mm^3^ and a viral load from a normal distribution with a mean of 4.46 log copies and standard deviation of 0.99 (**Table S4**).

**Table S2**. Probability distributions for initialization of CD4 count strata and VL strata within HIV infected compartments

|  |  | CD4 Count Category/Compartment (*c*) | | | | |
| --- | --- | --- | --- | --- | --- | --- |
|  |  | **0-50** | **51-200** | **201-350** | **351-500** | **>500** |
| Viral Load Category or Compartment (*v*) | **0-2.5** | 0.000000 | 0.000000 | 0.003968 | 0.003968 | 0.031746 |
|  | **2.5-3.5** | 0.000000 | 0.000000 | 0.012698 | 0.012698 | 0.073016 |
|  | **3.5-4.5** | 0.005952 | 0.017857 | 0.038095 | 0.046429 | 0.184524 |
|  | **4.5-5.5** | 0.013095 | 0.039286 | 0.061905 | 0.078571 | 0.178572 |
|  | **>5.5** | 0.016667 | 0.050000 | 0.059524 | 0.034524 | 0.036905 |

- - 1. Incorporation of equilibrium results from stochastic disease model into epidemic model

The rate of transition between these compartments ([*cd,v,m*]_(t)_ 🡪 [*cd,v,m*], _(t+1))_; ****_cd1v1m1cd2v2m2,_ hereafter shortened to **π_h_**) were determined by referencing rate transition tables under two different conditions (on ART and off ART) depending on ART threshold (assumed to be <200 cells/mm^3^ under base case). These rate transition tables were generated from the previously mentioned disease progression model.

This was achieved by using the previously mentioned Braithwaite stochastic HIV progression model to determine the rates of transition between CD4 count, viral load categories, and resistance categories and then substituting these calculated rates into a deterministic model. More specifically, separate one million trial simulations were conducted (under conditions of no ART available and ART available), to generate “off-care” and “on-care” estimates of disease progression. During these simulations state transitions between the aforementioned viral load categories, CD4 categories, and resistance categories were tracked, as well as transitions between any combination of these and HIV-related death. Rates were then calculated and “lookup tables” generated that indexed these state transitions by current CD4, VL, ART status, resistance category and HIV-related death (yes/no).

We evaluated the validity of this construction by subjecting the compartmental model to a similar analysis as that performed in a previous published analysis [2] using the Braithwaite stochastic model after harmonizing the assumptions and inputs between the two models. Results of the comparison between different monitoring strategies were similar between equivalent simulations (stochastic model vs. transmission model). In addition, the rank order of strategies from most effective to least was nearly identical between the two different models. We concluded, therefore, the deterministic model mimics the mean behavior of the stochastic model.

Progression through the spectrum of engagement was modeled as a stepwise dependent process starting from HIV uninfected through HIV infected and on ART (**Figure S1**). Initiation of ART was simulated when CD4 category fell beneath stated ART threshold and corresponded to the utilization of the “on care” integrated look-up table. Mortality from HIV (if infected) (μ*_h_*) could occur from any infected compartment while mortality unrelated to HIV (μ_age_) could occur from any compartment. Under conditions of treatment, transitions between CD4, VL strata, resistance strata are referred to as π’_h_ and AIDS related mortality rates as μ’_h_. HIV-related mortality rates (μ_h_ or μ’_h_) were determined from indexing the appropriate HIV progression model lookup tables.

1. **Initialization of population matrix**

We calculated the initial populations of each compartment in the model using census data from Maharashtra, India from 1991 (62,784,171) and 2001 (96,878,627), and interpolating to derive an estimate for 1997 (91,496,195), reflecting both the HIV uninfected and HIV infected population.[7] Maharashtra was chosen because the proposed clinical trial planned to take place in Mumbai, a major city in that state . Proportions of HIV infected individuals stratified by age and gender were determined from United Nations population data.[8] It was assumed that at the start of the simulation (i.e. 1997) that all HIV infected persons were in the undetected or detected compartments but none were in ART treatment compartments. We assumed that at the simulation start time, a HIV prevalence in the adult population of 0.3% and that none of infected persons had been tested and were aware of their results.[9] We did not assume any differences in likelihood of HIV testing between genders.

The simulation was run over an 18-year calibration period, representing the HIV epidemic during the period of 1997-2014. During this initialization period from 2003 onwards the baseline (i.e. assumed values for 1997) annual probability of undergoing HIV testing (0%) and the probability of linking to care given HIV infected (0%) were linearly scaled upwards to their assumed 2014 values of 2% and 30% respectively. This was incorporated into the model in order to represent the expansion of ART treatment programs within this setting. At the end of the initialization/calibration period a “snapshot” of the model population was captured and functioned as the initial population for all predictive/analytic simulations described in the manuscript. The initial population after this calibration period is outlined in **Table S3**. Distributions across sexual activity classes (*l*) can be calculated by multiplying a given cell with its respective probability conditional on gender.

**Table S3.** Calculated initial population distribution after calibration period

| Group | Susceptible | All HIV infected† | HIV infected and on ART†† |
| --- | --- | --- | --- |
| Children | 39,196,764 | 9,103 | 1,170 |
| Men | 30,919,916 | 114,611 | 61,240 |
| Women | 29,549,801 | 69,843 | 26,799 |
| Totals | 99,666,481 | 193,556 | 89,210 |

†Includes those HIV infected persons who are undetected (i.e. not tested), detected but not in care, and those in care and treatment programs (both on and off ART)

††Only those detected, in care and on ART

- 1. Entry and Maturation

Entry into the population was determined by the age-associated fertility rate for Maharashtra in 2008 and was distributed in relative proportion by risk group (p). Entry was assumed at birth but no sexual activity was assumed to occur until age 19. For any given compartment (not accounting for HIV infection or HIV-related death) entry, maturation and mortality were calculated as follows:

Newborns:

N_a=0,r_ (*t+1*) = Births*_r_* – ((1/D_a=0_) * N_a=0,r_ (*t*)) − μ_age_  (8)

Other age groups:

N_a,r_ (*t+1*) = ((1/D_a=0_)*N_a=0,r_ *(t)* )) – ((1/D_a_) * N_a,r_ *(t)* )) - μ_age_ (9)

Newborns acquire HIV through vertical transmission according to a probability that varies with the mother’s viral load. Mothers taking PMTCT meds are assumed to have a viral load two log strata lower than their actual viral load. (Ex. Women with a VL category of 5 will be attributed a vl category of 3 when determining the proportion of infected births.) Newborns are allocated equally across genders and are assigned into risk groups in the same proportions that adults are allocated. They are assigned CD4 and VL categories using the same allocation probabilities as adults who acquire new infection. Infected newborns inherit the resistance category of the mother.

1. **Representation of unhealthy alcohol consumption and a cognitive based therapeutic intervention to reduce its impact on HIV transmission and acquistion**

Based on a systematic review of pathways through which alcohol may impact HIV transmission risk unhealthy alcohol use was modeled as having three main effects: (1) increasing the risk of condom nonuse (RR 1.29 for unsafe sex [10,11]) (2) increasing the risk of ART non-adherence (RR 2.33 of missing doses based on pooled estimate from 4 studies [12–15]) and (3) increasing sexually transmitted infection (STI) prevalence (RR 1.72). [16,17]

As published previously[18], four population compartments were used to characterize alcohol use and the effects of the CBT intervention in the population: (1) Population targeted by intervention (includes all persons regardless of alcohol consumption) (2) unhealthy alcohol users, identified via screening, who have not yet been treated by an intervention aiming to reduce alcohol use, (3) unhealthy alcohol users who were successfully treated by an intervention and who now maintain healthy alcohol use, and (4) unhealthy alcohol users who were unsuccessfully treated by an intervention and who continue to misuse alcohol (**Figure S3**). The population within compartment (2) and (4) were modeled as having increased condom nonuse, ART non-adherence and STI prevalence associated with unhealthy alcohol use as discussed above.

In the alcohol intervention, cognitive behavior therapy was applied to only those who had unhealthy alcohol use and had not yet received an intervention (compartment 2). The intervention was either assumed to be available to all adults with unhealthy alcohol use or was further targeted to a subset of the hazardous drinkers based on their status (ex. Targeting only those who have HIV and are currently on treatment) and/or based on their viral load level (ex. Targeting only those with detectable viral load). In the same cycle the intervention is applied, unhealthy alcohol users are moved to either compartment 3 (successfully treated and now with healthy alcohol use) or compartment 4 (unsuccessfully treated and maintaining unhealthy alcohol use) as impacted by the assumed intervention effect size. Once in these treatment compartments, there is no movement out of them, with the assumption that there is a subset of the unhealthy alcohol group which is able to be cured through continual alcohol-based interventions and those that will continue hazardous drinking regardless of the intervention. Costs are continuously applied to all groups within the targeted population. The intervention cost was operationalized as an average cost for the intervention as a large proportion of individuals only receive the screening portion of the intervention while a smaller proportion of the population (i.e. those identified as unhealthy alcohol users receive the intensive therapy)) for each cycle. We addressed costs in this way because the increased complexity of the model that would be needed to account for differential cost based on services received was limited by computing resources and time.

**Figure S3. Schematic of alcohol intervention modeling**

**5 Outcomes**

The system of nonlinear differential equations were solved numerically to calculate the number of persons in each compartment over time. The following outcome measures were then derived.

5.1Effectiveness

5.1.1 Total number of HIV infections was calculated at a given time t as:

 Σ N _y>1_(t)

Where y refers to status variable (1=susceptible, >1 = HIV infected)

5.1.2 Prevalence at time t was calculated as:

 Σ N _y>1_(t) / Σ N(t)

5.1.3 Number of new infections over time horizon T was calculated as:

New infections= ∫^T^ Σ I_y>1_(t) * N_y=1_(t)dt [8]

5.1.4 Number of infections averted was calculated as:

# of new HIV infections (base case) - # of new HIV infections (intervention scenario)

- - 1. QALYs

The mean QALYs per year associated with each starting compartment was determined from the progression model and imported into the transmission model via the lookup tables. The total QALYs for each time cycle was calculated as the product of the number of people in each compartment multiplied by that compartment’s annual cost, summed over all compartments. The utility scores were stratified on CD4^+^ strata.[19]

5.2 Costs

Costs that were accounted for included acute hospitalization costs secondary to AIDS-related clinical events, 1^st^ and 2^nd^ line ART drugs, monitoring costs (varied by different monitoring strategy considered), and routine outpatient care of HIV infected persons. Costs were discounted at 3%. The mean annual cost associated with each starting compartment was determined from the progression model and imported into the transmission model via the lookup tables. The total cost for each time cycle is the product of the number of people in each compartment multiplied by that compartment’s annual cost, summed over all compartments.

**Table S4. Key input parameters to simulation model**

1. **Sexual risk characteristics**

| **Variable** | **Description** | **Value** | **Source** |
| --- | --- | --- | --- |
| **P_abs, k=2, o=1_** | Proportion of straight males who are abstinent | 27.75% | [20] |
| **Pr_l=2,k=2, o=1_** | Proportion of straight males who are in stable, monogamous relationships | 57.33% | [20] |
| **Pr_l=3,k=2, o=1_** | Proportion of straight males in multiple, concurrent relationships (Class 3) | 12.92% | [20] |
| **Pr_,l=2,k=2, o=1_** | Proportion of straight males in multiple, concurrent relationships (Class 4) | 2.0% | [21] |
| **P_abs, k=2, o=2_** | Proportion of bisexual males who are abstinent | 0% | -- |
| **Pr_l=2,k=2, o=2_** | Proportion of bisexual males who are in stable, monogamous relationships | 23.0% | [22] |
| **Pr_l=3,k=2, o=2_** | Proportion of bisexual males in multiple, concurrent relationships (Class 3) | 67.5% | Assumption |
| **Pr_,l=2,k=2, o=2_** | Proportion of bisexual males in multiple, concurrent relationships (Class 4) | 9.5% | [22] |
| **P_abs, k=2, o=3_** | Proportion of homosexual males who are abstinent | 0% | -- |
| **Pr_l=2,k=2, o=3_** | Proportion of homosexual males who are in stable, monogamous relationships | 23.0% | [22] |
| **Pr_l=3,k=2, o=3_** | Proportion of homosexual males in multiple, concurrent relationships (Class 3) | 67.5% | Assumption |
| **Pr_,l=2,k=2, o=3_** | Proportion of homosexual males in multiple, concurrent relationships (Class 4) | 9.5% | [22] |
| **P_abs, k=1, o=1_** | Proportion of straight females who are abstinent | 26.0% | [23] |
| **Pr_l=2,k=1, o=1_** | Proportion of straight females who are in stable, monogamous relationships | 69.0% | [23] |
| **Pr_l=3,k=1, o=1_** | Proportion of straight females in multiple, concurrent relationships (Class 3) | 4.7% | [20] |
| **Pr_l=3,k=1, o=1_** | Proportion of straight females in multiple, concurrent relationships (Class 4) | 0.3% | [22] |
| **P_abs, k=1, o=2_** | Proportion of bisexual females who are abstinent | 26.0% | [22] |
| **Pr_l=2,k=1, o=2_** | Proportion of bisexual females who are in stable, monogamous relationships | 69.0% | [23] |
| **Pr_l=3,k=1, o=2_** | Proportion of bisexual females in multiple, concurrent relationships (Class 3) | 4.7% | [23] |
| **Pr_,l=2,k=1, o=2_** | Proportion of bisexual females in multiple, concurrent relationships (Class 4) | 0.3% | [20] |
| **P_abs, k=1, o=3_** | Proportion of homosexual females who are abstinent | 26.0% | [22] |
| **Pr_l=2,k=1, o=3_** | Proportion of homosexual females who are in stable, monogamous relationships | 69.0% | [23] |
| **Pr_l=3,k=1, o=3_** | Proportion of homosexual females in multiple, concurrent relationships (Class 3) | 4.7% | [23] |
| **Pr_,l=2,k=1, o=3_** | Proportion of homosexual females in multiple, concurrent relationships (Class 4) | 0.3% | [20] |

1. **Sexual transmission**

| **Variable** | **Description** | **Value** | **Source** |
| --- | --- | --- | --- |
| **α^s^_k=2,K=1_** | Transmission risk per sex act (F🡪M) | 0.00042 | [24] |
| **α^s^_k=1,K=2_** | Transmission risk per sex act (M🡪F) | 0.00081 | [24] |
| **α^s^_k=2,K=2_** | Transmission risk per sex act (M🡪M) | 0.00169 | [24] |
|  |  |  |  |
| **α^m^_v=0_** | Relative risk of transmission if VL category 0-2.5 log copies/ml | 0.16 | [25] |
| **α^m^_v=1_** | Relative risk of transmission if VL category 2.5-3.5 log copies/ml | 1.87 | [25] |
| **α^m^_v=2_** | Relative risk of transmission if VL category 3.5-4.5 log copies/ml | 6.54 | [25] |
| **α^m^_v=3_** | Relative risk of transmission if VL category 4.5-5.5 log copies/ml | 8.85 | [25] |
| **α^m^_v=4_** | Relative risk of transmission if VL category >5.5 log copies/ml | 9.03 | [25] |
| **ε** | Degree of assortative mixing between age and sexual activity classes (0=assortative, 1=proportionate) | 0.20 | [4] |
| **d_l=1_** | Average duration (years) of stable, monogamous partnerships | 30.0 | Assumption |
| **d_l_** | Average duration (years) of partnership in activity group 2 | 1.0 | Assumption |
| **d_l=3_** | Average duration (years) of partnership in activity group 3 | 0.5 | Assumption |
| **r_l=1_** | Median number of concurrent partnerships for activity group 1 | 1.0 | Assumption used in [18] |
| **r_l=2_** | Median number of concurrent partnerships for activity group 2 | 3.0 | Assumption used in [18] |
| **r_l=3_** | Median number of concurrent partnerships for activity group 3 | 10.0 | Assumption used in [18] |

1. **HIV risk behaviors and biological/behavioral modifiers of transmission**

| **Variables** | | **Description** | **Value** | **Source** |
| --- | --- | --- | --- | --- |
| **P_STI_** | Prevalence of untreated STI | | 6% | [26] |
| **P_circ_** | Probability of not being circumcised | | 80% | [27] |
| **P_alc_** | Initial proportion unhealthy alcohol use, males | | 18.5% | [28] |
|  | Initial proportion unhealthy alcohol use females | | 20% | + |
| **P_condom_** | Prevalence of condom nonuse most or all of the time | | 73% | [23] |
|  | Relative risk of alcohol/mental health/drug use on condom nonuse | | 1.29 | [10,11] |
|  | Relative risk of alcohol/mental health/drug use on ART nonadherence | | 2.33 | [12–15] |
|  | Relative risk of alcohol/mental health/drug use on having an untreated STI | | 1.72 | [16,17] |
| **A^p^_condom_** | Relative risk reduction of HIV seroconversion when using condoms | | 80% | [29] |
| **A^p^_STI_** | Relative risk reduction of HIV seroconversion if treated STI | | 40% | [30] |
| **A^p^_circ_** | Relative risk reduction of HIV seroconversion if circumcised | | 59% | [31] |
| **α_IDU_** | Transmission risk per injection | | 0.0036 | [32] |
| **P_IDU_** | Proportion of population that uses IV drugs | | 4.99E-5 | [22] |
| **P_IDU-risky_** | Proportion of IDU with unsafe injection practices | | 32% | Assumption used in [33] |
|  | Number of needle sharing partners per year | | 5 | [34] |
|  | Shared injections per year | | 102 | [22] |

+Based on survey responses from our patient population (unpublished)

| **Variable** | **Description** | **Values** | **Source** |
| --- | --- | --- | --- |
| **P_test_** | Probability/rate of not being tested for HIV | 98% | NACO 2014 |
| **P_LTC_** | Probability of linkage to care once HIV diagnosed | 30% | [35] |
|  | Mean CD4 count (SD) for newly infected individuals | 644 (260) | [36] |
|  | Mean log viral load (SD) for newly infected females | 4.46 (0.99) | [36] |
|  | CD4 count threshold for treatment initiation | 200 | [37] |
| **Padh** | Probability of nonadherence | 26% | [38] |
| **** | Fertility rate (range, depending on age) | 0.0027-0.23 | [8] |
|  |  |  |  |
| **Costs** |  | **2014 USD** |  |
|  | 1^st^ line ART monthly costs | $11.86 | [39] |
|  | 2^nd^ line ART monthly costs | $49.27 | [39] |
|  | Annual routine costs if in HIV care and treatment program | $132.18 | [19] |
|  | Annual inpatient hospitalization costs if patient has AIDS | $347.25 | [19] |
|  | HIV-1 viral load test | $49.54 | [39] |
|  | CD4+ count test | $6.32 | [39] |

1. **HIV disease related and Demographics**
2. **Calibration of HIV epidemic model**

We pre-specified four calibration criteria in order to evaluate whether the model’s predictions were compatible with observed results: HIV prevalence, HIV incidence, proportion of people with HIV on treatment and the annual proportion of people who have died from HIV (**Figure S4**). We compared our model output compared to India-specific data spanning over 1997-2013 from UNAIDS. [40]

**Figure S4 Calibration of India HIV Simulation Model.**

a, Comparing model prevalence results with reported data for India. b, Comparing model incidence results with reported data for India. c, comparing annual proportion of people dying of HIV in model with reported data for India. d, comparing proportion of people with HIV on treatment compared with reported data for India.

**Technical Appendix References**

1. Braithwaite RS, Justice AC, Chang C-CH, Fusco JS, Raffanti SR, Wong JB, et al. Estimating the proportion of patients infected with HIV who will die of comorbid diseases. Am. J. Med. 2005;118:890–8.

2. Braithwaite RS, Nucifora KA, Yiannoutsos CT, Musick B, Kimaiyo S, Diero L, et al. Alternative antiretroviral monitoring strategies for HIV-infected patients in east Africa: opportunities to save more lives? J. Int. AIDS Soc. 2011;14:38.

3. Garnett GP, Anderson RM. Balancing sexual partnerships in an age and activity stratified model of HIV transmission in heterosexual populations. IMA J. Math. Appl. Med. Biol. 1994;11:161–92.

4. Garnett GP, Anderson RM. Factors controlling the spread of HIV in heterosexual communities in developing countries: patterns of mixing between different age and sexual activity classes. Philos. Trans. R. Soc. Lond. B. Biol. Sci. 1993;342:137–59.

5. Morgan D, Rutebemberwa A, Malamba S, Ross A, Whitworth J, Kaleebu P, et al. HIV-1 RNA levels in an African population-based cohort and their relation to CD4 lymphocyte counts and World Health Organization clinical staging. J. Acquir. Immune Defic. Syndr. 1999. 1999;22:167–73.

6. Saathoff E, Pritsch M, Geldmacher C, Hoffmann O, Koehler RN, Maboko L, et al. Viral and host factors associated with the HIV-1 viral load setpoint in adults from Mbeya Region, Tanzania. J. Acquir. Immune Defic. Syndr. 1999. 2010;54:324–30.

7. Mahārāshtra (India): State, Major Agglomerations & Cities - Population Statistics in Maps and Charts [Internet]. [cited 2016 Aug 29]. Available from: http://www.citypopulation.de/India-Maharashtra.html

8. World Population Prospects - Population Division - United Nations [Internet]. [cited 2016 Aug 29]. Available from: https://esa.un.org/unpd/wpp/

9. HIV and AIDS in India | AVERT [Internet]. [cited 2016 Aug 29]. Available from: http://www.avert.org/professionals/hiv-around-world/asia-pacific/india

10. Weiser SD, Leiter K, Heisler M, McFarland W, Percy-de Korte F, DeMonner SM, et al. A population-based study on alcohol and high-risk sexual behaviors in Botswana. PLoS Med. 2006;3:e392.

11. Weiser SD, Leiter K, Bangsberg DR, Butler LM, Percy-de Korte F, Hlanze Z, et al. Food insufficiency is associated with high-risk sexual behavior among women in Botswana and Swaziland. PLoS Med. 2007;4:1589–97; discussion 1598.

12. Byakika-Tusiime J, Crane J, Oyugi JH, Ragland K, Kawuma A, Musoke P, et al. Longitudinal antiretroviral adherence in HIV+ Ugandan parents and their children initiating HAART in the MTCT-Plus family treatment model: role of depression in declining adherence over time. AIDS Behav. 2009;13 Suppl 1:82–91.

13. Nakimuli-Mpungu E, Bass JK, Alexandre P, Mills EJ, Musisi S, Ram M, et al. Depression, alcohol use and adherence to antiretroviral therapy in sub-Saharan Africa: a systematic review. AIDS Behav. 2012;16:2101–18.

14. Nduna M, Jewkes RK, Dunkle KL, Shai NPJ, Colman I. Associations between depressive symptoms, sexual behaviour and relationship characteristics: a prospective cohort study of young women and men in the Eastern Cape, South Africa. J. Int. AIDS Soc. 2010;13:44.

15. Tadios Y, Davey G. Antiretroviral treatment adherence and its correlates in Addis Ababa, Ethiopia. Ethiop. Med. J. 2006;44:237–44.

16. Chersich MF, Luchters SMF, Malonza IM, Mwarogo P, King’ola N, Temmerman M. Heavy episodic drinking among Kenyan female sex workers is associated with unsafe sex, sexual violence and sexually transmitted infections. Int. J. STD AIDS. 2007;18:764–9.

17. Fisher JC, Cook PA, Sam NE, Kapiga SH. Patterns of alcohol use, problem drinking, and HIV infection among high-risk African women. Sex. Transm. Dis. 2008;35:537–44.

18. Kessler J, Ruggles K, Patel A, Nucifora K, Li L, Roberts MS, et al. Targeting an alcohol intervention cost-effectively to persons living with HIV/AIDS in East Africa. Alcohol. Clin. Exp. Res. 2015;39:2179–88.

19. Freedberg KA, Scharfstein JA, Seage GR, Losina E, Weinstein MC, Craven DE, et al. The cost-effectiveness of preventing AIDS-related opportunistic infections. JAMA. 1998;279:130–6.

20. Nag M. Sexual behaviour in India with risk of HIV/AIDS transmission. Health Transit. Rev. 1995;5:293–305.

21. NACO. India: Annual Report 2012-2013. 2013.

22. National AIDS Control Organization (NACO), Ministry of Health and Family Welfare, Government of India. NACO, Ministry of Health and Family Welfare, Government of Indi; 2009.

23. National AIDS Control Organization (NACO), Ministry of Health and Family Welfare, Government of India. New Delhi, India: NACO, Ministry of Health and Family Welfare, Government of Indi; 2006.

24. Boily M-C, Baggaley RF, Wang L, Masse B, White RG, Hayes RJ, et al. Heterosexual risk of HIV-1 infection per sexual act: systematic review and meta-analysis of observational studies. Lancet Infect. Dis. 2009;9:118–29.

25. Attia S, Egger M, Müller M, Zwahlen M, Low N. Sexual transmission of HIV according to viral load and antiretroviral therapy: systematic review and meta-analysis. AIDS Lond. Engl. 2009;23:1397–404.

26. World Health Organization. Global Prevalence and Incidence of Selected Curable Sexually Transmitted Infections Overview and Estimates. 2001;

27. UNAIDS. Male circumcision. Global trends and determinants of prevalence, safety and acceptability. 2007;

28. Obot SI, Room R. Alcohol, Gender and Drinking Problems. World Health Organ. Dep. Ment. Health Subst. Abuse Geneva. 2005;

29. Weller S, Davis K. Condom effectiveness in reducing heterosexual HIV transmission. Cochrane Database Syst. Rev. 2002;CD003255.

30. Grosskurth H, Mosha F, Todd J, Mwijarubi E, Klokke A, Senkoro K, et al. Impact of improved treatment of sexually transmitted diseases on HIV infection in rural Tanzania: randomised controlled trial. Lancet Lond. Engl. 1995;346:530–6.

31. Gray RH, Wawer MJ, Brookmeyer R, Sewankambo NK, Serwadda D, Wabwire-Mangen F, et al. Probability of HIV-1 transmission per coital act in monogamous, heterosexual, HIV-1-discordant couples in Rakai, Uganda. Lancet Lond. Engl. 2001;357:1149–53.

32. Tokars JI, Marcus R, Culver DH, Schable CA, McKibben PS, Bandea CI, et al. Surveillance of HIV infection and zidovudine use among health care workers after occupational exposure to HIV-infected blood. The CDC Cooperative Needlestick Surveillance Group. Ann. Intern. Med. 1993;118:913–9.

33. Kessler J, Myers JE, Nucifora KA, Mensah N, Kowalski A, Sweeney M, et al. Averting HIV Infections in New York City: A Modeling Approach Estimating the Future Impact of Additional Behavioral and Biomedical HIV Prevention Strategies. PLOS ONE. 2013;8:e73269.

34. Mahanta J, Medhi GK, Paranjape RS, Roy N, Kohli A, Akoijam BS, et al. Injecting and sexual risk behaviours, sexually transmitted infections and HIV prevalence in injecting drug users in three states in India. AIDS Lond. Engl. 2008;22 Suppl 5:S59–68.

35. Sarna A, Sebastian M, Bachani D, Sogarwal R, Battala M. Pretreatment loss-to-follow-up after HIV diagnosis from 27 counseling and testing centers across India: findings from a cohort study. J. Int. Assoc. Provid. AIDS Care. 2014;13:223–31.

36. Mehendale SM, Bollinger RC, Kulkarni SS, Stallings RY, Brookmeyer RS, Kulkarni SV, et al. Rapid disease progression in human immunodeficiency virus type 1-infected seroconverters in India. AIDS Res. Hum. Retroviruses. 2002;18:1175–9.

37. AmPath [Internet]. [cited 2016 Sep 14]. Available from: http://ampath.com/

38. Allam RR, Murhekar MV, Bhatnagar T, Uthappa CK, Chava N, Rewari BB, et al. Survival probability and predictors of mortality and retention in care among patients enrolled for first-line antiretroviral therapy, Andhra Pradesh, India, 2008-2011. Trans. R. Soc. Trop. Med. Hyg. 2014;108:198–205.

39. Walensky RP, Ross EL, Kumarasamy N, Wood R, Noubary F, Paltiel AD, et al. Cost-effectiveness of HIV treatment as prevention in serodiscordant couples. N. Engl. J. Med. 2013;369:1715–25.

40. Epidemiology publications | UNAIDS [Internet]. [cited 2016 Jun 15]. Available from: http://www.unaids.org/en/dataanalysis/knowyourepidemic/epidemiologypublications/
